# Clinical advances and challenges of antibody-mediated targeted drug delivery in breast cancer therapeutics

**DOI:** 10.1007/s12672-026-04492-5

**Published:** 2026-02-01

**Authors:** Mokhtar Rejili

**Affiliations:** Department of Biology, College of Sciences, Imam Mohammad Ibn Saud Islamic University (IMSIU), 11623 Riyadh, Saudi Arabia

**Keywords:** Antibody–drug conjugates, Drug delivery systems, Nanoparticles, Precision medicine, Targeted therapy

## Abstract

Breast cancer (BC) continues to present a universal health burden, and thus there is a need to develop antigen-targeted therapies with lower off-target toxicity and a greater therapeutic index. The antibody-mediated targeted drug delivery systems are especially antibody–drug conjugates (ADCs) and antibody-conjugated nanoparticles (ACNPs) have become the game changers in the age of precision oncology. By taking advantage of the selectivity of monoclonal antibodies, these platforms preferentially target tumor-associated antigens (TAAs) including HER2, TROP-2, HER3 and LIV-1, which avoids off-target toxicity and overcomes drug resistance. This review presents a detailed discussion of the principles of design, mechanisms and clinical advancements of ADCs and ACNPs in the treatment of BC. Major advances are in the field of advanced linker technology, site selective conjugation techniques, and novel payloads that have potent antitumor effects. A number of new-generation ADCs have demonstrated promising clinical results, especially in HER2-positive and triple-negative forms of the BC. Moreover, nanocarrier-based drug delivery systems like trastuzumab-functionalized liposomes or polymeric nanoparticles are more advantageous in drug loading and release, as well as tumor penetration. In spite of this progress there are still several challenges including heterogeneity of tumor antigens, immunogenicity and off-target toxicity which hinder clinical translation. Bispecific antibodies, dual-payload ADCs, and patient-specific antigen profiling are some of the strategies under research to improve the precision of therapeutic treatment. With the application of molecular targeting and drug delivery innovation, the biology of antibody-mediated systems has enormous potential to transform the standard of treatment of BC and pave the way to customised medicine. Antigen selection, rational linker-payload design, and biomarker-directed patient stratification. In BC, ADC-led antibody-mediated delivery is setting therapeutic delivery action, and after all, toxicity management and mitigation of resistance are core clinical factors in success.

**Graphical Abstract:**

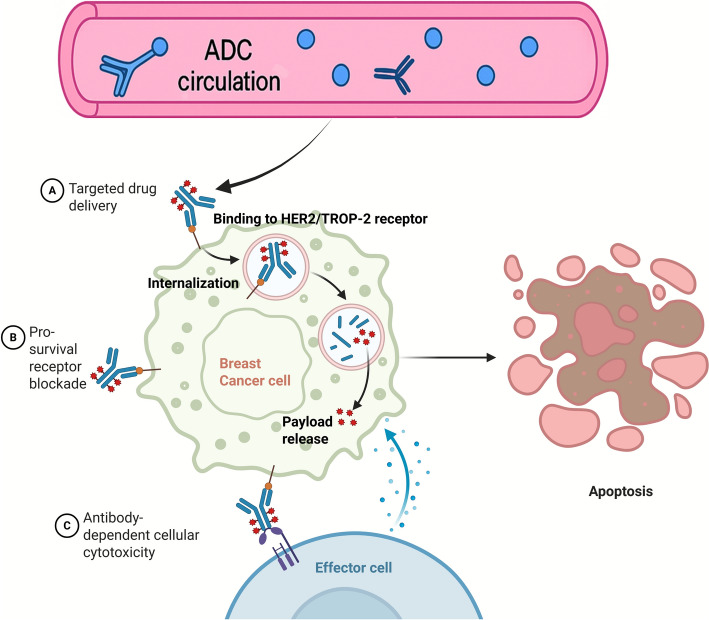

**Supplementary Information:**

The online version contains supplementary material available at 10.1007/s12672-026-04492-5.

## Introduction

Breast cancer (BC) is the most frequently diagnosed cancer and a leading cause of cancer-related death amongst women globally [1]. In line with the estimates in the Global Cancer Observatory (GLOBOCAN) 2022, BC has more than 2.3 million new cases and about 685,000 deaths in every corner of the world, highlighting its high prevalence among both population and health systems (and its vast impact on healthcare) [2]. Although screening methods and early detection have improved, a significant number of patients receive a late diagnosis and necessitate intensive and lengthy systemic therapy [3].

BC is a biologically heterogeneous disorder that is conventionally described in terms of hormone receptor expression (estrogen receptor [ER] and progesterone receptor [PR]) and human epidermal growth factor receptor 2 (HER2) [4]. The classification leads to the occurrence of major molecular subtypes, such as luminal A (ER+/PR+/HER2−), luminal B (ER+/PR+/HER2+), HER2-enriched (ER−/PR−/HER2+), and triple-negative breast cancer (ER − /PR −/HER2−) [5, 6]. All subtypes have specific prognostic characteristics, treatment responses, and clinical results. An example is the common association of HER2+ and TNBC with aggressive disease progression, poor prognosis, and unresponsiveness to treatment [7].

Traditional chemotherapy is still one of the pillars of BC treatment. Nevertheless, its non-selective mechanism of action causes systemic toxicity, immune-suppression, gastrointestinal side effects, and hair loss, hindering the quality of life of the patients considerably [8, 9]. Besides, the emergence of multidrug resistance (MDR) through efflux pumps such as P-glycoprotein, and changes in drug metabolism, adds to therapeutic failure and recurrence of the disease [10]. These restrictions have led to a pressing necessity to have more selective and effective treatment approaches. Whereas active in most settings, standard regimens tend to be restricted to non-selective exposure of tissues that cause myelosuppression, neuro-toxicity, gastrointestinal toxicity, alopecia, and dose-restricted response, and encourage relapse and unfavorable response durability. Comparisons of the antibody-based systems are quicker in amplifying the therapeutic index by directing the cytotoxins to the tumor-associated antigens and sparing normal tissues [11].

Targeted therapy has become a revolution in the field of oncology with the aim of improving treatment accuracy and reducing systemic toxicity. Antibody-mediated targeted drug delivery systems (DDS) have been receiving significant attention considering that they are highly specific totumor-associated antigens (TAAs). Even monoclonal antibodies (mAbs) can be designed to be able to target specific surface markers that are largely expressed on cancer cells [12], allowing cytotoxic agents to be delivered directly to the tumor site [13, 14]. This accuracy minimizes harm to healthy tissues and provides the possibility to defeat the mechanisms of resistance to drugs [15]. Examples are trastuzumab emtansine (T-DM1) in the case of HER2+ BC that incorporates both the HER2-targeting properties of trastuzumab and the cytotoxic effects of DM1 [16].

Due to the increasing clinical significance of antibody–drug conjugates (ADCs) and the changing environment of targeted therapeutics, there is an urgent need to summarize existing information on antibody-mediated DDS development, utilization, and prospects in BC. ADCs are engineered with both high-affinity targeting and powerful payload and (with cleavable linkers) generate a beneficial bystander effect in nonhomogeneous lesions [17]. Antibody-conjugated nanoparticles (ACNPs) are polymerized with additional benefits, increased drug dosage, controlled or stimulated release and active targeting through antibody decoration which increase deposition into the tumor and reduce systemic exposure [18]. Taken together, the features help to address major deficiencies of standard chemotherapy, and are consistent with the objectives of precision-oncology in BC. This review aims to provide a comprehensive overview of the scientific principles, design strategies, clinical progress, and challenges associated with these therapies. Furthermore, we highlight recent innovations and emerging platforms that promise to enhance therapeutic efficacy and specificity, ultimately contributing to the personalized treatment of BC. To be clear, ADCs are classified as clinically established ones, and ACNPs are described as investigational platforms, with no FDA approval at the moment; their addition is dedicated to the opportunities of translational and further directions, but not to the approved practice.

## Principles of antibody-mediated drug delivery

**Fig. 1:**
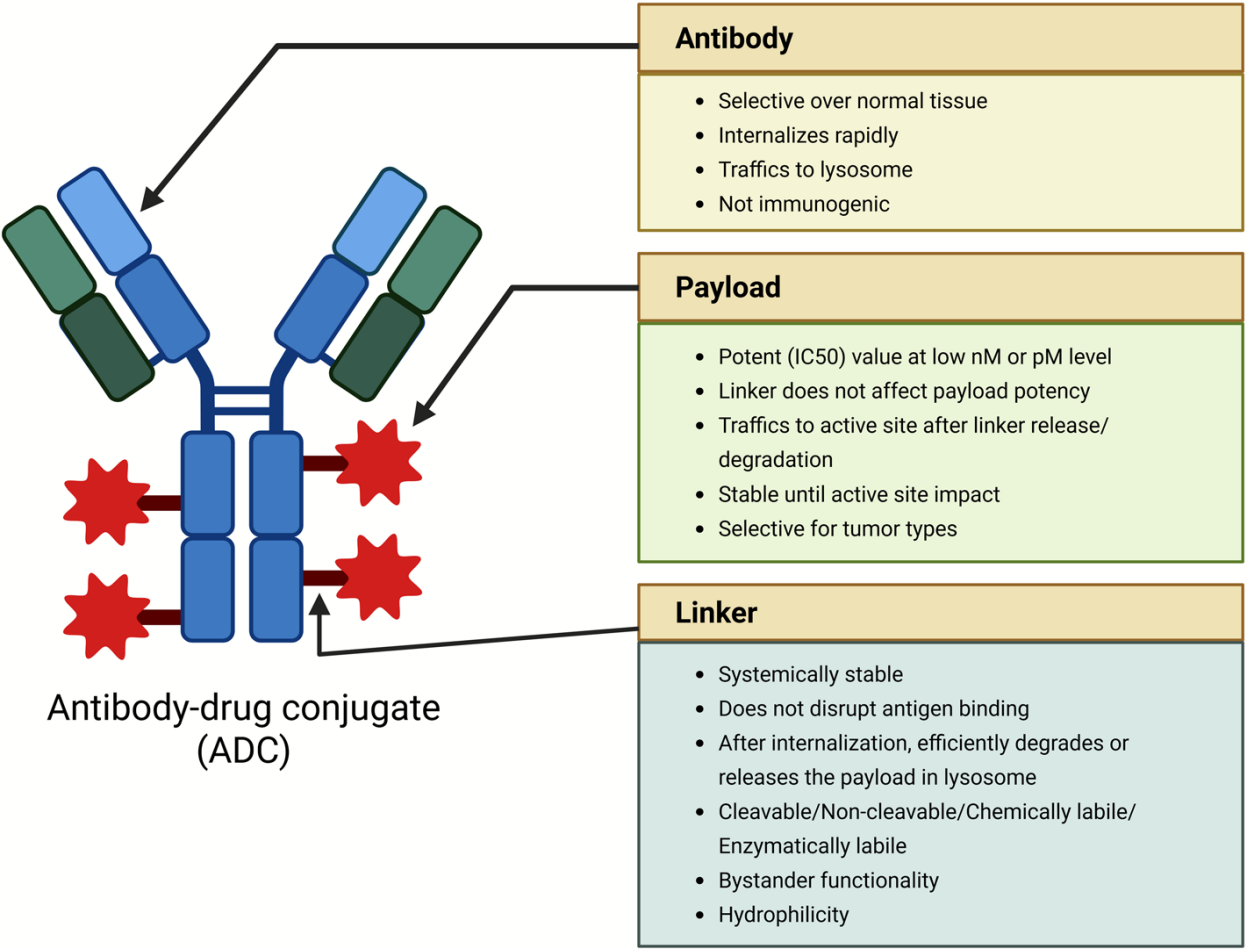
Structure of Antibody–Drug Conjugates (ADCs)

mAbs or antibody fragments are chemically linked via a cleavable or non-cleavable linker to a cytotoxic chemical (payloads), such as cytotoxic drugs. This forms strong ADCs comprising of the antibody, the linker and the payload (Fig. 1). They are very effective anti-tumor reagents and can be used to provide chemotherapy drugs to cancer cells in a highly targeted and direct manner against the specific cells under strict regulation of antibodies with high specificity and affinity [19, 20]. Table 1 presents a summary of the Antibody-mediated drug-delivery systems in BC.

**Table 1:** Antibody-mediated drug-delivery systems in breast cancer (Overview of Sect. 2)

System/component	Defining features	Typical target antigens in BC	Representative payload classes or cargo	Release/activation paradigm	Key advantages	Principal challenges in BC	Representative examples/status
Monoclonal antibodies	High-affinity antigen recognition; Fc-mediated effector functions	HER2, EGFR, MUC1, GPNMB, TROP-2*	– (no cytotoxic payload)	Receptor blockade; ADCC/CDC	Target specificity; validated biomarkers	Limited efficacy in low-antigen tumors; resistance via signaling bypass	Trastuzumab, pertuzumab (approved)
ADCs	Antibody + linker + cytotoxic payload	HER2, TROP-2, others under evaluation	Microtubule inhibitors (DM1, MMAE), Topo-I inhibitors (DXd, SN-38), DNA-damaging (PBDs)	Cleavable (acid-labile, disulfide, enzyme-cleavable) or non-cleavable (thioether); bystander effect depends on linker/payload	High therapeutic index; delivers potent drugs to cancer cells	Antigen heterogeneity; on-target off-tumor toxicity; interstitial penetration; resistance (efflux, lysosomal processing)	T-DM1, trastuzumab deruxtecan; sacituzumab govitecan (approved)
Antibody-conjugated nanoparticles (ACNPs)	Antibody-decorated liposomes/polymeric NPs; active targeting	HER2, EGFR, MUC1, etc	Chemo payloads (e.g., doxorubicin, paclitaxel), siRNA/miRNA, combinations	Triggered release (pH, enzyme, redox) and EPR + active binding	High drug loading; controlled/triggered release; multi-cargo potential	Scale-up/reproducibility; protein corona; heterogeneity of EPR; translation	Anti-HER2 immunoliposomes; investigational ACNPs (clinical/late preclinical)
Linkers	Connect Ab to payload; govern stability & release	–	–	Cleavable: acid-labile (hydrazone), disulfide, enzyme-cleavable (cathepsin-B peptides); Non-cleavable: thioether (SMCC)	Tuneable stability; enables bystander effect (cleavable)	Premature cleavage/systemic toxicity vs. over-stability/low release	See Supplementary Table S1 (chemistries & examples)
Payloads	Ultra-potent cytotoxins suited for intracellular release	–	MMAE/DM1 (microtubules), DXd/SN-38 (Topo-I), PBDs (DNA)	Endosomal/lysosomal release; diffusion if membrane-permeable	High potency at low DAR	Bystander toxicity (membrane-permeable), efflux, payload-specific AEs	Payloads as used in approved ADCs
Mechanism Targeting → internalization → endolysosomal processing	Antigen-mediated uptake; trafficking dictates release	–	–	Linker cleavage or proteolysis; possible extracellular release (bystander)	Selective intracellular delivery	Requires sufficient antigen density and internalization kinetics	–
Antigen selection	Tumor-associated, high density, internalizing	HER2, TROP-2, EGFR, MUC1, GPNMB	–	–	Improves selectivity; guides patient selection	Heterogeneous expression; normal-tissue expression	HER2 & TROP-2 clinically validated targets

In the following paragraphs, we outline the different types of ADCs relevant to BC, emphasizing their components, mechanisms, and clinical applications.

### mAbs

The antibodies with high antigen specificity compared to non-target proteins are one of the most important elements of ADC design. Low specificity antibodies cross reacting with other antigens can develop unexpected effects. They might also react with healthy tissues, resulting in off-target toxicities or get eliminated before the treatment site [20]. Previous ADCs were susceptible to immune responses because of mouse antibodies, and thus humanized antibodies were invented which comprise a mixture of human sequences with the mouse antigen-binding segments. Even with these developments, immune response may still occur, inspiring current studies on human (or engineered) antibodies. There is even an emergence of new formats such as bispecific antibodies (bsAbs), which must be evaluated regarding immunogenicity carefully [21].

Among the five groups of immunoglobulins (IgA, IgM, IgE, IgG, and IgD), IgG is the most preferable option in ADCs because it is abundant in the body, maintaining a stable structure, and less immunogenic [22]. Its Fc component contributes to its stability to allow safe introduction of DDS into cancer cells where it is able to induce cell death. The capacity of IgG to readily access cancer cells makes it the best compound upon which to base the development of ADCs to combat malignancies. These characteristics render IgG as the best antibody to deliver very high toxic loads in ADCs tailored to address cancer cells [23, 24].​ The reason preference is on its IgG1 subclass because it has long serum half-life in the blood relative to smaller sized antibody fragments, enabling it to be exposed to target cells longer. It activates immunogenic vectors, such as ADDC and CDC, increasing its anti-tumor effect of cytotoxic drug delivery [25, 26]. The number and location of available reactive handles, rather than IgG subclass itself, are the main determining factor of the achievable drug-to-antibody ratio (DAR) with each of them potentially influencing its conjugation ability and stability, yet the concept of a given IgG subclass does not inherently imply a higher payload capacity. Nevertheless, IgG3 even with its high tumor-killing ability is eliminated in the body too fast [27]. Conversely, IgG4 has the ability to form half-bodies, and this might have a special role of creating hybrid molecules of the type of bispecific immunoglobulins [13]. The second most immunoglobulin in abundance to IgG is IgA, which plays a very important role in mucosal immunity and that has the potential of attacking mucosal cancers as well. It has been found to exhibit attractive characteristic features that render it a promising therapeutic candidate, although its production and purification have been a problem because of complicated glycosylation [20, 28]. Large immunoglobulin IgM is capable of targeting low antigen-expressing cancer cells, which boosts the ability of ADC to induce cancer cell death in some cancers. Its large size and sophisticated framework, however, create difficulties in the conjugation and internalization of drugs [29]. Recently, IgM-ADC was generated, which is selectively conjugated at the C-terminus manufacturing designed selenocysteines on each heavy chain to treat chronic lymphocytic leukemia [30, 31]. Because of its ability to stimulate other immune cells in addition to IgG, IgE is and is currently being investigated as an immunotherapy for cancer because it offers an alternative method of targeting tumors, particularly solid tumors. Its primary difficulty is reducing its allergic reactions but maintaining the anti-tumor activity, which has resulted in the development of safer IgE-based ADCs [32, 33]. IgD has a short half-life due to its sensitivity to protease and quick degradation. Though it plays a limited role in immune responses, researchers are exploring its potential in ADC development. More studies are needed to understand its therapeutic applications [34]. The current drug targeting by antibody has developed the use of site-specific conjugation technologies (click chemistry, enzymatic ligation) that provide consistent drug: antibody ratios and enhanced stability. Tumor selectivity and antigen escape are overcome by bispecific and biparatopic antibodies, which increase tumor selectivity. Novel Fc engineering and glyco-optimization techniques to enhance circulation half-life and immunogenicity, and nanobody fragments and nanobody conjugation to enhance tumor penetration and targeted DDS [35].

### Linkers

**Fig. 2:**
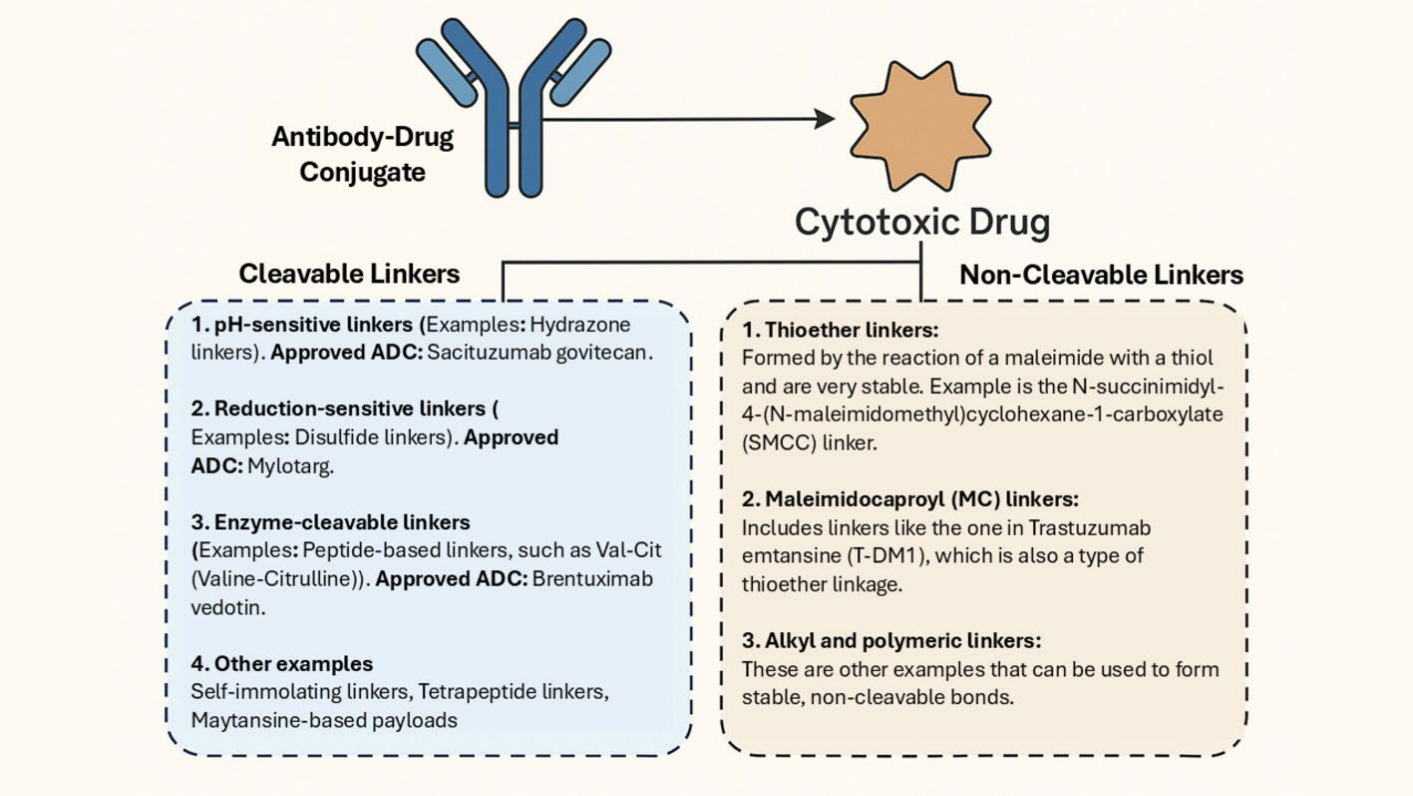
Classification of Linkers in Antibody–Drug Conjugates (ADCs). The diagram illustrates the two main classes of linkers used in ADCs; cleavable and non-cleavable. Cleavable linkers include hydrazone, disulfide, peptide, and β-glucuronide-based linkers, which release the cytotoxic drug in response to specific intracellular stimuli such as acidic pH, reducing agents, or enzymatic activity. Non-cleavable linkers, such as thioether and amide bonds, rely on the complete degradation of the antibody within the lysosome for drug release

Linkers play a critical role in creating a connection between the mAb and the cytotoxic payload, making them highly adaptable components of an ADC. They significantly influence the biophysical and functional characteristics of the ADC, including its stability, potency, effectiveness, and toxicity. Furthermore, linkers serve the important functions of preventing the premature release of the cytotoxic drug in the bloodstream and ensuring the efficient release of the drug at the target site [36]. Linkers can be broadly classified into two classes based on the release mechanisms of the payloads (Fig. 2): cleavable (Hydrazone Bond, Disulfide Bond, Peptide bond, and β-Glucuronide linker) and non-cleavable (thioether bond and amide bond) which are explained below in detail [37]. The Antibody-linker-payload circulates intact; Cleavable linkers are triggered in endo/lysosomes, while non-cleavable linkers rely on lysosomal proteolysis of the antibody; and a self-immolative spacer collapses to release the active payload.

#### Cleavable linkers

Cleavable linkers are preferred over non-cleavable linkers in ADCs because they offer better control over when and where the cytotoxic drug is released. Cleavable linkers are designed to remain stable in the bloodstream but break down when exposed to the specific conditions found within cancer cells, such as acidic environments, the presence of proteases, or reducing agents. Typically, the acidic conditions that trigger cleavage correspond to endosomal or lysosomal compartments with pH values ranging from ≈4.5 to 6.0, compared with the near-neutral extracellular or plasma pH of ≈7.4, allowing selective hydrolysis of acid-labile linkers such as hydrazone or cis-aconityl bonds. There are reduced chances of off-target toxicity and it will also be possible to deliver the drug to cancer cells mainly without harming normal tissues; thereby maximizing the therapeutic effect and minimizing the damage to the healthytissues [38]. Cleavable linkers can be classified into two groups: chemically cleavable (can be acid-labile and disulfide linkers, cleavable by exogenous stimuli), and enzyme-cleavable (linkers with dipeptide sequences, that is, β-glucuronidase-cleavable and phosphatase-cleavable linkers).

**Chemically cleavable** Acid labile linkers: Acid-cleavable hydrazone linkers are relatively stable at physiological pH (~ 7.4) but not completely; they undergo slow hydrolysis with low-level payload release over 24–48 h, in contrast to rapid cleavage in acidic endosomal/lysosomal compartments (pH ~ 4.5–6) [37]. This was demonstrated in early trials of Pfizer's gemtuzumab ozogamicin (Mylotarg), which uses an acid-sensitive N-acyl hydrazone linker. In acidic conditions, the linker hydrolyzes to release the cytotoxic drug, ensuring effective DDS [39].

Disulfide linkers: Disulfide linkers are commonly used in ADCs because they are stable at physiological pH but degrade in the presence of thiols inside cells. These linkers have proven effective in clinical ADCs like Pfizer's Mylotarg and Besponsa [37]. Traditional aliphatic disulfide linkers do not survive in human plasma; they are subject to thiol-disulfide interchange with the plasma thiols especially the free Cys34 residue of the human serum albumin with the threat of premature payload release. Proven in clinical designs thus rely on chemically-modified disulfides, e.g., sterically-hindered tertiary/aryl disulfides, disulfide masking or self-immolative structures, to increase plasma stability whilst maintaining intracellular-reducibility. In contrast, high levels of glutathione (GSH) in cancer cells enable selective breakdown of these linkers, leading to drug release and making them effective for targeting cancer cells [40]. Disulfide linkers are often paired with maytansinoid payloads can be readily conjugated to the linker component via appropriate functional handles. This makes maytansinoids ideal for ADCs that deliver potent drugs directly into tumor cells [41]. The sulfur-containing handles of the maytasinoids are amenable to disulfide coupling, and upon the catabolism process of these molecules by the cell, will result in membrane-permeable catabolites allowing them to achieve a bystander effect in heterogeneous tumors. Why it is not universal: (i) due to the overriding interests of non-cleavable linkers to reduce bystander toxicity and enhance plasma stability; (ii) peptide linkers or alternative designs a better fit to PK, safety and efficacy can make disulfides suboptimal; and (iii) disulfides may be suboptimal because of target biology and clinical intent. Disulfide-maytansinoid pairing will therefore not be a default rule, but site-specific.

Cleaving by exogenous stimuli: Using external triggers to break ADC linkers improves drug release control, accommodating biological differences. Stenton et al. created a thioether-based linker that releases its drug upon exposure to a palladium catalyst, showing similar effectiveness to Dox with a HER2-targeting nanobody. However, safer palladium complexes are required for clinical applications [42, 43]. Rossin et al. developed a trans-cyclooctene (TCO) linker that is cleaved by a tetrazine-triggered reaction. Using pegylated tetrazine, they achieved 90% drug release in 20 h, outperforming enzyme-cleavable ADCs in mice. These advancements highlight the potential of external triggers for more precise and effective ADC treatments [42].

**Enzyme cleavable linker** Dipeptide-containing linkers: Enzymes, such as cathepsin, which are active in tumor cells, could be used to cleave the linkers containing dipeptides, which would ensure that the drugs were released primarily within the target cells and reduced the side effects [44]. Valine-citrulline (Val-Cit) dipeptide linker is mostly cleaved by cathepsin B, which is an overexpressed enzyme in most tumors. Val-Cit-PABC remains stable in the plasma of human beings until cathepsin B cleaves off in lysosomes. Ces1c (carboxylesterase-1c) can cleave the linker/PABC prematurely in mice, resulting in these mice having an apparent instability unless Ces1c-knockout mice are used. This difference in species may be taken into account whenever extrapolating preclinical PK/PD and toxicity [45]. The cleavage not only releases cytotoxic agents; its selectivity is also low because cathepsin activity in normal tissues is low, which has made Val-Cit linkers attractive in ADCs such as brentuximab vedotin (Adcetris). The other example is phenylalanine-lysine (Phe-Lys) linker that is based on protease cleavage as well. These dipeptide conjugates can circulate effectively and cleave effectively within tumors thus represented an efficient balance between stability and specificity [46]. Dipeptide linkers are attractive to ADCs as they take advantage of tumor-specific protease activity and permit targeted drug release resulting in a small number of effects on normal tissues. Continued studies are currently going on to improve their stability and efficiency to better therapeutic results.

β-Glucuronidase-cleavable linkers: These linkers utilize the enzyme β-glucuronidase, active in lysosomes and secreted in necrotic tumor areas, allowing ADCs to target both extracellular and intracellular environments. First explored for prodrugs in 1988, they were adapted for ADCs by Seagen Inc. (formerly Seattle Genetics) in 2006. Combined with a para-aminobenzylcarbamate (PABC) spacer, these linkers demonstrated impressive stability and efficacy in preclinical studies [47]. PABC spacer, a self-immolative linker element that, after protease cleavage of the upstream peptide, spontaneously fragments to release the free active payload [48]. Compared to traditional dipeptide linkers, β-glucuronidase-cleavable linkers offer better plasma stability and lower aggregation, even at a high DAR. This decreased aggregation can be attributed to hydrophilic/polar linker architectures in which the overall hydrophobicity of the ADC increases with higher DAR hence reducing inter-antibody hydrophobic interactions. Also, self-immolative spacers and site-directed conjugation approaches can be utilized to create solubility and colloidal stability as the payloads are solubilized and spread off of Fc surfaces. These features combined provide great-DAR designs which have reasonable PK and manufacturability.

These properties enhance the effectiveness of ADCs in vivo and reduce plasma clearance due to increased hydrophilicity. Adding PEG chains further improves ADC exposure and activity, making high-DAR ADCs (DAR = 8) feasible without affecting pharmacokinetics. This technology shows promise for improving ADC stability, loading capacity, and therapeutic effectiveness [37, 49].

β-galactosidase-cleavable linkers: β-Galactosidase-cleavable linkers exploit lysosomal β-galactosidase to unmask a galactose-caged spacer and release payload intracellularly. Preclinical studies demonstrate potent activity and in vivo efficacy of galactoside-linked ADCs, but published head-to-head comparisons versus cathepsin-cleavable Val-Cit ADCs in mouse models are limited/absent; therefore, any superiority claims should be considered unproven [50, 51]. A 3-O-sulfo-β-galactose linker that is sequentially cleaved by sulfatase → β-galactosidase shows excellent selectivity and cytotoxicity in HER2-targeted ADCs (preclinical) [45].

**Other enzyme-responsive linkers**

Phosphatase-triggered phosphate/phosphoramidate cages have been explored preclinically for bioconjugates and prodrugs, but they are not clinically validated for ADCs and remain far less established than peptide (cathepsin-cleavable) or β-glucuronide motifs. We therefore do not treat them as a core class here [52, 53].

#### Non-cleavable linkers

 Non-cleavable linkers form stable bonds that are not enzymatically or chemically cleaved in the tumor milieu; payload release occurs only after lysosomal degradation of the antibody, yielding a payload-linker catabolite that retains activity. Non-cleavable designs can reduce bystander effects and improve plasma stability, with efficacy dependent on the activity of the resulting catabolite. In non-cleavable designs, the ADC must be completely proteolyzed in lysosomes, to produce a charged, membrane-impermeable catabolite. Since this catabolite cannot diffuse through membranes, the cytotoxic response is highly focused on the antigen-positive cell that was infected by the ADC, and the cells surrounding it are not subjected to it, i.e., there is no bystander effect. Conversely, cleavable linkers are cleavable to release membranepermeable payloads which can diffuse passively into cells adjacent to the cell releasing cleavable linkers, to exert a bystander effect. Implication: non-cleavable linkers have been shown to work best in antigen-homonous tumors, and cleavable/by-stander-competent ADCs could be more effective regarding heterogeneous antigen expression. Additionally, cleavable linkers are more versatile and adaptable to different tumor environments, making them a more flexible option in ADC development. Their design helps ensure that the drug is only released once the ADC has successfully entered the cancer cell, improving both efficacy and safety [54]. The main advantage of non-cleavable linkers compared to cleavable ones is their better stability in the bloodstream, which leads to lower off-target toxicity and improves the overall safety and tolerability of the ADCs. However, non-cleavable ADCs can be less effective against tumors because they lack the "bystander effect" that helps kill surrounding cancer cells, especially when the target antigen is not evenly distributed [55, 56]. Some non-cleavable linkers are the following:

Maleimide linkers: Maleimide linkers selectively react with thiol groups (-SH) via a Michael addition, forming stable thioether bonds for precise attachment of the cytotoxic payload to the antibody. This site-specific conjugation allows for effective control of the DAR, optimizing ADC efficacy while minimizing potential toxicity [57].

Amide and amino acid linkers: Amide linkers are integral to ADCs due to their stability and non-cleavable properties. They create robust bonds between the drug and antibody, ensuring retention until lysosomal degradation. While they prevent premature release, the resulting drug-linker conjugate may exhibit reduced potency due to its attachment to the antibody [58]. Amide and amino acid-based linkers share similar cleavage mechanisms, primarily relying on enzymatic hydrolysis and tumor microenvironment sensitivity to achieve controlled drug release. Amino linkers in ADCs utilize amino groups (-NH2) for drug attachment, offering flexibility as either cleavable or non-cleavable options. Engineered for specific conditions like enzymatic cleavage or pH changes, these linkers ensure effective DDS to cancer cells while minimizing harm to healthy tissues [59].

Hydrophilic linkers: Hydrophilic linkers enhance the solubility and pharmacokinetics of ADCs by incorporating elements like polyethylene glycol (PEG) or sugars. This design improves circulation in the bloodstream, reduces off-target toxicity, and ensures effective DDS to tumors, particularly in ADCs with high DAR [60].

### Payload

An important component of an ADC is its payload which has good cancer-killing effects once it is delivered into the tumor cell. Payloads should be highly potent to be effective, i.e. IC50 values should be between 01 and 0.1 nm. They are also required to be stable within the body and have functional groups which can chemoselectively couple into the antibody through a suitable linker [54, 61]. The three broad categories of modern ADC payloads are the microtubules-targeting types of auristatins and maytansinoids; the DNA-cleavage-targeting calicheamicins; and the topoisomerase-1-inhibitory camptothecins. These act either by disrupting DNA, inducing apoptosis or by destabilizing the activity of microtubules, resulting in G2/M phase arrest and cell death. The microtubule-targeting agents are the most widely employed in the ADCs, which makes use of the rapid division of cancer cells. Microtubule-destabilizing payloads, such as auristatins, maytansinoids, disrupt microtubule dynamics through binding to the vinca/maytansine site of β-tubulin to inhibit polymerization and thus mitotic arrest; microtubule-stabilizing agents include taxanes [56, 62].

The more recent derivatives of auristatin, such as MMAE and MMAF, enhance stability and activity, and developments in the maytansinoid group, such as hydrophilic linkers and derivatives including DM1 and DM4, have served to overcome difficult-to-treat patients, such as MDR [63]. A range of microtubule-targeting agents such as tubulysins, cryptophycins, and hemiasterlin interfere with cell division. DNA-damaging payloads, including calicheamicins and duocarmycins, are highly effective with a mechanism of action that involves killing cancer cells by causing DNA breakages, alkylation, and cross-linking, at lower antigen concentrations [64]. New payloads such as tubulysins, cryptophycins, dual payloads, and immune-stimulating delivery vehicles are being demonstrated to have enhanced antitumor effect with side effects minimally sensitive to toxicity in preclinical models and in the initial phase of clinical trials, albeit context-dependent [65–67].

## Mechanism and antigen selection in antibody-mediated drug delivery

### ADC mechanisms: targeting, internalization, and release

**Fig. 3:**
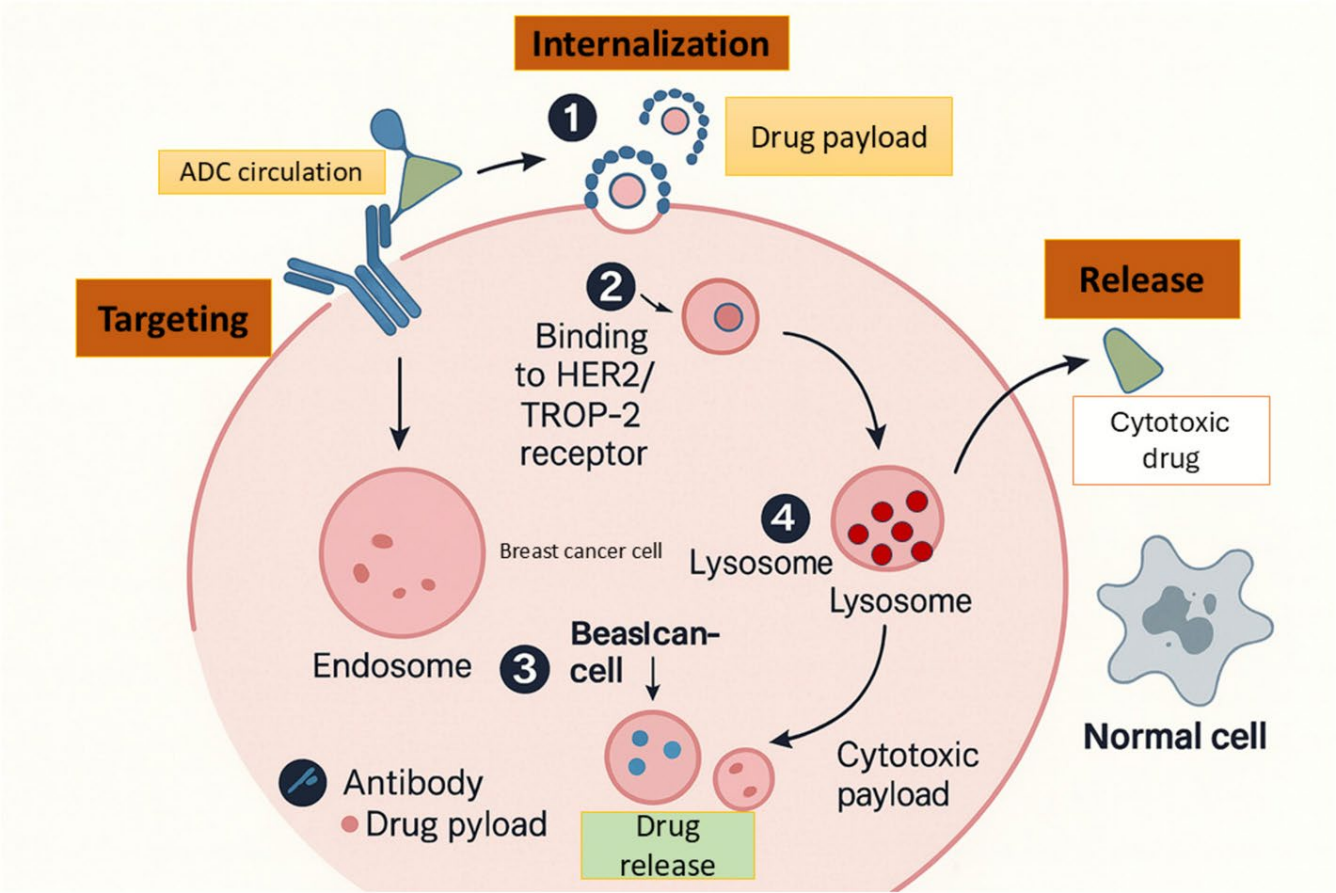
Mechanism of targeted cytotoxic drug delivery and cellular internalization pathways. The targeting moiety (e.g., antibody or ligand) binds to a specific receptor on the cell surface, facilitating cellular uptake via various endocytic routes such as clathrin-mediated endocytosis, caveolae-independent pathways, CLIC/GEEC pathways, and macropinocytosis. Once internalized, the drug-carrier complex traffics to the lysosome, where the cytotoxic drug is released. The released drug induces cell death, highlighting the effectiveness of targeted therapeutic delivery systems in cancer treatment

ADCs aim to transport potent cytotoxic agents to cancerous cells with the minimal impact on the normal tissues. Upon the administration, ADCs are carried throughout the blood, seek particular antigens on the surface of the cancer cells. Following an ADC binding to its antigen target, an ADC-antigen complex is formed, which is internalized by the tumor cell via endocytosis in three steps, (1) bud formation, (2) membrane curvature and vesicle maturation and (3) scission and release of the membrane into the cytoplasm (Fig. 3) [68]. ADC internalization regulates two major endocytosis pathways, including Clathrin-Mediated Endocytosis (CME), and Clathrin-Independent Endocytosis. The proteins of clathrin create a coated pit in the cell membrane, which in CME is used to bend and cluster the receptors. Dynamin also pinches the pit to form a clathrin-coated vesicle (CCV). The CCV dissolves into endosomes and ADCs are broken down [69]. In Clathrin-Independent Endocytosis, several pathways are involved, such as Caveolae-Mediated Endocytosis employing small pits named caveolae, but very few buds on the membrane restricting the uptake of ADC [70]. It also involves the CLIC/GEEC Pathway and Macropinocytosis, but their functions in ADC internalization have been less established, and are in research [71]. A degradation process then occurs in which the ADC is transported to a lysosome, where it is degraded and the cytotoxic drug is released into the cell of the tumor. The released drug may impact in numerous ways, including interrupting the replication of DNA or disrupting the functionality of microtubules, and eventually, leading to the death of the tumor cell. Signaling pathway activation to cause apoptosis or production of reactive oxygen species (ROS) leading to oxidative stress are other mechanisms of action under investigation [72].

For ADCs to be effective, the target antigen must be primarily expressed on cancer cells, and support efficient internalization; quantitative analyses suggest a practical lower bound of roughly ~ 10,000 receptors per cell for many targets, although the exact threshold is antigen- and payload-dependent [73]. The antibody should have high specificity, and affinity, and be non-immunogenic. The cytotoxic payload needs to be highly potent, as only 1–2% reaches the target inside the cell. Optimizing the DAR and using novel cytotoxic agents are key to improving ADC efficacy, making them a promising cancer treatment [74, 75].

### Selection of tumor-specific antigens

The success of antibody-mediated targeted DDS in BC hinges on the precise identification of tumor-specific or TAAs (TSAs/TAAs). Ideal targets should demonstrate high expression levels onmalignant cells with minimal presence in healthy tissues to minimize off-target toxicity. In BC, several antigens including HER2, Trophoblast Cell Surface Antigen 2 (Trop-2), Epidermal Growth Factor Receptor (EGFR), Mucin 1 (MUC1), and Glycoprotein Non-Metastatic Melanoma Protein B (GPNMB) have been explored extensively due to their overexpression and involvement in oncogenic processes.

These are the key types of tumor-specific/associated antigens relevant to BC (including lineage-specific receptors, differentiation antigens, cancer-testis antigens, overexpressed surface glycoproteins and stromal/ECM-associated targets) and the key ones, which include the selection criteria, heterogeneity of expression, and clinical relevance, are discussed in the following paragraphs. A detailed and updated summary of tumor-specific antigens in breast cancer, including recent clinical examples, has been provided as Supplementary Table S1.

#### HER2

HER2 is overexpressed in approximately 15–20% of BC and is associated with aggressive tumor phenotypes and poor prognosis. As a member of the ErbB family, HER2 lacks a known ligand but forms heterodimers with other family members to drive downstream signaling involved in cell proliferation and survival [76, 77]. HER2-targeted therapies such as trastuzumab, pertuzumab, and ADC T-DM1 have transformed the clinical management of HER2-positive BC [78]. However, primary and acquired resistance, as well as cardiotoxicity, remain significant challenges [79]. New-generation ADCs such as trastuzumab deruxtecan (T-DXd) have shown promise by leveraging a high DAR and a cleavable linker that enables a potent bystander effect [80].

#### Trop-2 (Trophoblast Cell Surface Antigen 2)

Trop-2 is a transmembrane glycoprotein involved in cell proliferation and survival, and it is overexpressed in various epithelial cancers including TNBC [81]. Its restricted expression in normal tissues and high surface density on tumor cells makes it an attractive target. Sacituzumab govitecan (SG), a Trop-2–directed ADC conjugated to SN-38 (the active metabolite of irinotecan), has demonstrated efficacy in metastatic TNBC, receiving FDA approval in 2020 [82]. However, dose-limiting toxicities such as neutropenia and diarrhea underscore the need for strategies to enhance selectivity and minimize systemic exposure [83].

#### EGFR (Epidermal growth factor receptor)

EGFR is overexpressed in up to 50% of TNBCs and plays a role in promoting proliferation, angiogenesis, and resistance to apoptosis. While EGFR has been a validated target in non-small cell lung cancer and colorectal cancer, its translation to BC has been more complex due to heterogeneous expression and resistance mechanisms [84, 85]. Clinical trials of EGFR inhibitors (e.g., cetuximab) in BC have shown limited benefit as monotherapies but may offer synergy when combined with chemotherapeutic agents or immune checkpoint inhibitors (ICIs) [86]. ADCs targeting EGFR, such as depatuxizumab mafodotin, are also under investigation.

#### MUC1 (Mucin 1)

MUC1 is a glycoprotein normally expressed on the apical surface of epithelial cells, but in cancer, it becomes overexpressed and aberrantly glycosylated, exposing tumor-associated epitopes. This altered form is frequently present in over 90% of BC, making it a prime candidate for targeted delivery systems [87]. Challenges include variability in glycoform expression across tumor subtypes and the risk of targeting normal tissues expressing MUC1. Several MUC1-directed ADCs and immunotherapies are in preclinical or early clinical development, such as MUC1-C-specific agents and bsAbs [88].

#### GPNMB (Glycoprotein non-metastatic melanoma protein B)

GPNMB is a type I transmembrane protein implicated in cell invasion, metastasis, and angiogenesis, particularly in TNBC and basal-like BC subtypes. It is minimally expressed in normal tissues, except skin and melanocytes, making it a suitable therapeutic target [89, 90]. Glembatumumab vedotin, an ADC targeting GPNMB, demonstrated encouraging results in early-phase trials but failed to show significant benefit in the METRIC phase IIb trial for TNBC [91]. These outcomes underscore the importance of patient stratification and antigen validation. Wider platform-level issues and future ADC strategies, such as penetration constraints, heterogeneity of antigens, resistance driven changes, and elaborate designs of linkers/payload/format are summarized in Sect. 3.3.

### Major challenges and next-generation ADC strategies in breast cancer

Regardless of the transformative activity of various ADCs in BC, there are numerous clinical and translational challenges which restrict consistent and lasting benefit. First, the antigen heterogeneity as well as a variable internalization decreases the concentration of payload and favors resistant subclones; even the low or heterogeneous expression of the target may be targetable with membrane-permeable payloads and cleavable linkers which induce the bystander effect, but again augment off-tumor risk [82, 91, 92]. Second, abnormal vasculature, increased interstitial fluid pressure, hypoxia and the high hydrodynamic radius of ADCs cause specific barriers in solid tumor malignant cells, preventing deep penetration; dose limits due to payload toxicities further limit tumor targeting [93, 94]. Third, resistance mechanisms are antigen loss/down-modulation, impaired trafficking/lysosomal processing, efflux pump upregulation, and payload-specific adaptations [95].

In practice, HER2-directed T-DXd showed better progression-free survival (PFS) and overall survival (OS) than the physician-chosen chemotherapy in the case of HER2-low metastatic disease, which enlarged the treatment population group, presenting the problem of interstitial lung disease (ILD) that needs close attention [92, 96]. TPROP-2-informed ADC SG increased survival in already treated metastatic triple-negative BC, and in the updated studies strengthened benefit and neutropenia with diarrhea as critical unwanted effects [82, 97, 98]. However, the gpNMB-targeted glembatumumab vedotin provided mixed/negative randomized results regardless of bio marker enhancing, which highlights the role of solid antigen choice and biology [91, 99].

Conjugation design next-generation ADC/Ab-conjugate design aims at (i) site-specific conjugation to tune DAR and stability; (ii) optimised cleavable linkers to balance stability with bystander activity; (iii) Topo-I and DNA-interactive payloads to overcome tubulin resistance; (iv) smaller scaffolds (e.g. sdAb-based or biparatopic antibodies) to enhance penetration; (v) bispecific/dual-payload ADCs to address heterogeneity; and All these methods are intended to increase the therapeutic index and overcome failures observed in some single-antigen programs.

## Antibody-mediated targeted drug delivery system for breast cancer treatment

ADCs are an advanced form of targeted therapy that integrates mAbs with potent cytotoxic agents. In cancer treatment, particularly oncology, ADCs are engineered to selectively transport these highly toxic compounds to malignant cells that express specific surface antigens, such as HER2. This precision approach aims to enhance therapeutic efficacy while minimizing off-target effects commonly associated with systemic chemotherapy. Owing to the remarkable therapeutic potential of ADCs, this area of research has rapidly gained momentum, resulting in a substantial increase in the number of ADCs developed and approved for clinical use over the last ten years [100]. Two major strategies have emerged in this context: ADCs and ACNPs. The current section focuses on describing these two approaches in detail. This sub-topic summarises ADCs, some of which have been approved by the FDA to be used as therapeutic agents in BC and other cancer types, and then varies to ACNPs as research tools, specifically, as antibody-mediated carriers that are under preclinical or early clinical trials.

### Antibody–drug conjugates

The development of ADCs was inspired by the “magic bullet” concept proposed for precision oncology; wherein therapeutic agents are selectively delivered to tumor cells while sparing normal tissues [101]. ADCs are typically composed of three essential components: a mAb specific to the tumor-associated antigen, a highly cytotoxic payload (also referred to as the “warhead”), and a linker that connects the antibody to the drug [102]. Once administered, the ADC circulates through the bloodstream and accumulates at the tumor site, where the antibody moiety binds specifically to the surface antigen on the cancer cell. This antigen–antibody complex is then internalized into the cell via endocytosis. Within the acidic lysosomal environment, the linker is cleaved, often through proteolysis, leading to the intracellular release of the cytotoxic agent. The active drug then disrupts key cellular processes such as DNA replication or microtubule function, ultimately inducing tumor cell apoptosis.

In cases where the payload is relatively non-polar, the released drug can diffuse across the cell membrane and exert effects on neighboring tumor cells, resulting in what is termed the “bystander effect.” This mechanism enables ADCs to retain cytotoxic efficacy even in tumors with low or heterogeneous expression of the target antigen [103]. Overall, ADCs constitute a promising class of therapeutics in oncology by enabling selective delivery of powerful cytotoxins to malignant cells. This section provides an overview of ADCs targeting key antigens such as HER2, TROP-2, HER3, and LIV-1 (Table S1). Illustrative examples include the non-cleavable thioether SMCC linker used in T-DM1, the enzyme-cleavable tetrapeptide (GGFG) linker used in T-DXd, and the acid-labile carbonate (CL2A) linker used in SG. These chemistries respectively exemplify lysosomal degradation-dependent release (SMCC), cathepsin-mediated cleavage with a self-immolative spacer (GGFG-PABC), and pH/esterase-facilitated hydrolysis (CL2A). Table 2 shows the ongoing and near-completion antibody-based therapies in BC.

**Table 2:** Ongoing and near-completion antibody-based therapies in breast cancer

Agent (Modality)	Target	Key trial/ID	Phase & status (as of Oct 2025)	Population / line	Selected notes
*Datopotamab deruxtecan (Dato-DXd; ADC)*	TROP-2	TROPION-Breast01 (NCT05104866)	Phase 3 primary results reported; PFS ↑ vs ICC; OS mixed; development ongoing	HR + /HER2-, post-CT	Positive primary analysis published 2025; ongoing regulatory/label work (PubMed)
*Datopotamab deruxtecan (ADC)*	TROP-2	TROPION-Breast02	Phase 3 (TNBC) OS benefit announced	1L mTNBC (IO-ineligible)	Press release: OS ↑ and PFS ↑ vs chemo (Oct 19, 2025) (AstraZeneca US)
*Patritumab deruxtecan (HER3-DXd; ADC)*	HER3	HERTHENA-Breast04	Phase 3 initiated (Aug 27, 2025)	Metastatic HR + /HER2- after ET	Builds on phase-2 activity incl. CNS/LMD cohorts (Merck)
*Zanidatamab (bispecific mAb)*	HER2 (2 ECDs)	Multiple phase 2 programs	Ongoing (final/updated readouts in 2024–25)	HER2 + and HER2-low mBC (various combos)	Emerging activity with chemo/evorpacept; continued evaluation in mBC (ASC Publications)
*Trastuzumab duocarmazine (SYD985; ADC)*	HER2	TULIP (NCT03262935)	Phase 3 PFS benefit; FDA CRL (2023); program updates ongoing	Pretreated HER2 + MBC	Phase 3 positive; ocular AEs notable; regulatory path evolving (PubMed)
*Disitamab vedotin (RC48; ADC)*	HER2 (incl. HER2-low)	RC48 programs (e.g., NCT05831878; NCT05904964)	Phase 2–3 ongoing	HER2-expressing / HER2-low ABC	Real-world and trial signals in HER2-positive/-low; randomized studies underway (OUP Academic)
*ARX788 (site-specific ADC)*	HER2	ACE-Breast-02/03	Seamless phase 2/3 program with interim/phase-3 reports	HER2 + (post-trastuzumab; T-DXd-pretreated cohorts)	Pivotal comparisons vs lapatinib + capecitabine; additional settings in progress (Nature)
*BNT323 (trastuzumab pamirtecan; ADC)*	HER2	China phase 3 vs T-DM1	Phase 3 met PFS (2025)	Pretreated HER2 + mBC	First phase-3 success for BioNTech oncology; broader programs planned (Reuters)

#### HER2-targeting system

HER2-directed ADCs have emerged as an important therapeutic option for BC patients whose tumors overexpress the HER2 receptor. HER2 is a transmembrane tyrosine kinase receptor involved in the regulation of cell growth and division. ADCs that target HER2 typically incorporate a mAb that specifically recognizes and binds to HER2 on the surface of tumor cells. Trastuzumab, a well-established HER2-targeting mAbs, is frequently used as the antibody component in these ADCs. It serves as a delivery vehicle to transport the attached cytotoxic agent directly to HER2-positive tumor cells [100].

**Trastuzumab emtansine (T-DM1)** T-DM1 was the first ADC approved for the treatment of BC. It combines the HER2-targeting capabilities of trastuzumab with DM1, a maytansine derivative that disrupts microtubule function, linked through a non-cleavable thioether linker [100]. Clinical data from Verma et al. (2012) demonstrated that T-DM1 significantly prolonged OS when compared to the combination of lapatinib and capecitabine, with a median OS of 30.9 months versus 25.1 months, respectively (p < 0.001) [104]. The EMILIA trial, a randomized, open-label, phase III clinical study, further confirmed the survival benefit of T-DM1. Results were consistent with those of Verma et al., with a median OS of 29.9 months for the T-DM1 group compared to 25.9 months in the control group [16].

In addition to its efficacy in advanced BC, T-DM1 has been explored for early-stage disease. The KATHERINE trial investigated the use of T-DM1 in patients with HER2-positive early BC who had residual invasive disease following neoadjuvant therapy. The study found that patients treated with T-DM1 had significantly higher invasive disease-free survival rates at 3 years compared to those receiving trastuzumab alone (88.3% vs. 77.0%, p < 0.001). These findings led to the U.S. FDA expanding the approval of T-DM1 for the adjuvant treatment of early-stage HER2-positive BC in 2019 [105, 106]. As a result, T-DM1 is currently approved for use in the adjuvant setting for patients with residual disease following neoadjuvant treatment with taxanes and trastuzumab-based therapy [100].

**Fig. 4:**
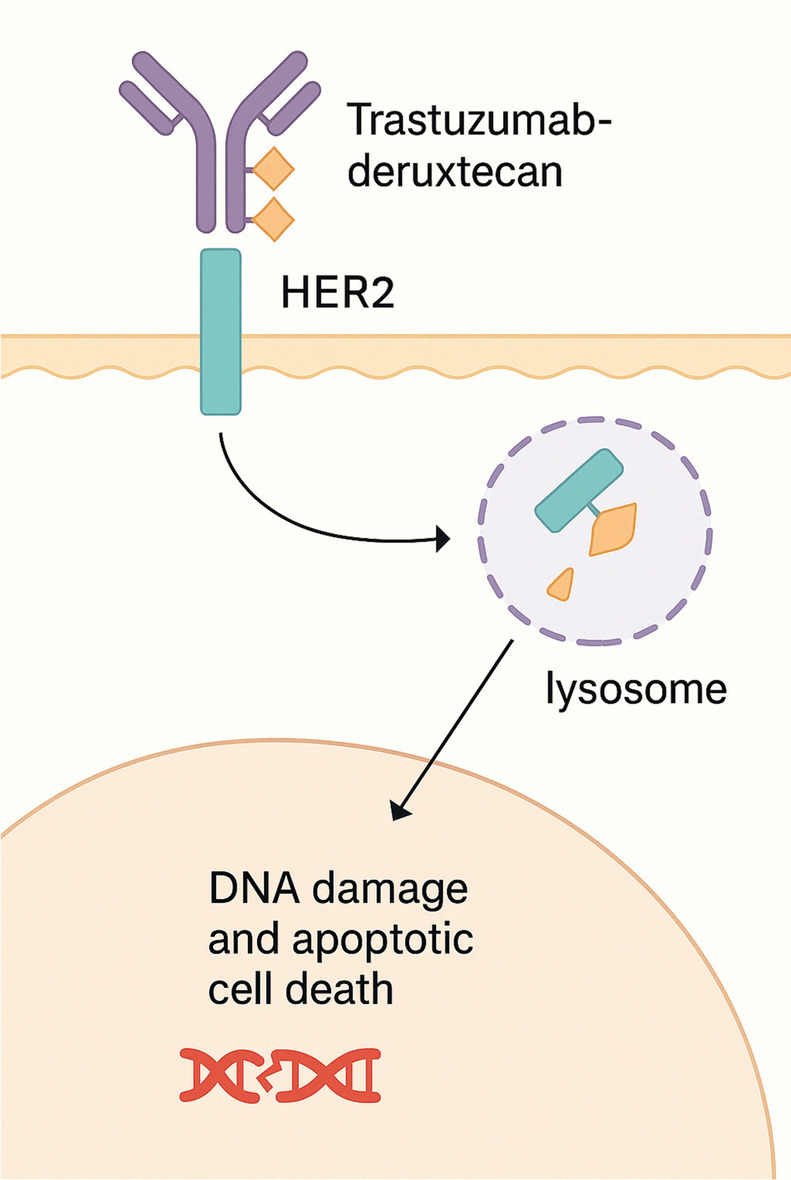
Mechanism of action of trastuzumab deruxtecan (T-DXd) in HER2-positive cancer cells. T-DXd binds to the HER2 receptor, leading to internalization of the antibody–drug conjugate. Following lysosomal degradation, the cytotoxic payload (Dxd) is released, inducing DNA damage and triggering apoptotic cell death in the nucleus

**Trastuzumab deruxtecan (T-DXd)** T-DXd is an advanced HER2-directed ADC that has gained regulatory approval for treating HER2-expressing BC and gastric cancers. It was the first ADC to be evaluated in a tumor-agnostic trial involving patients with various solid tumors expressing HER2 (Fig. 4). In these studies, T-DXd showed an impressive objective response rate (ORR), particularly among patients with immunohistochemistry (IHC) 3 + expression, along with sustained therapeutic effects and a tolerable safety profile in heavily pretreated cohorts [107].

Unlike T-DM1, T-DXd employs a different cytotoxic payload—an exatecan derivative (DX-8951f), which functions as a topoisomerase I inhibitor. Specifically, T-DXd employs a GGFG tetrapeptide linker incorporating a p-aminobenzyloxycarbonyl (PABC) self-immolative spacer. After internalization, lysosomal cathepsins cleave the GGFG sequence, triggering PABC self-immolation and release of free DXd. Following strong preclinical efficacy and favorable safety data, T-DXd progressed rapidly into clinical trials. The phase III DESTINY-Breast03 trial assessed the efficacy and safety of T-DXd and demonstrated significant improvements in PFS compared to T-DM1, along with a manageable toxicity profile. These findings led to its approval as a second-line therapy for HER2-positive metastatic BC [108].

**Trastuzumab duocarmazine (SYD985)** Trastuzumab duocarmazine (SYD985) or vic-trastuzumab duocarmazine is a third generation HER2-targeting ADC. Although it still contains the trastuzumab antibody conjugate it contains a duocarmycin analog hydrocarbon attached through a cleavable linker. SYD985 exhibited good efficacy and tolerability in early-phase clinical trials in patients with metastatic BC of HER2-positive type [109]. Consequently, it was granted the fast-track status by the U.S. FDA. The open-label phase III ratio trial identified SYD985 versus physician choice treatment (randomized) in patients with unresectable, locally advanced, or metastatic HER2-positive BC who had received at least two HER2-targeted therapies or T-DM1. SYD985 also postponed disease progression considerably. Yet, ocular toxicities were more common leading to increased discontinuation rate [110].

**Disitamab vedotin (RC48)** RC48-ADC is a HER2-targeting ADC comprised of the mAb disitamab conjugated with a cytotoxic agent monomethyl auristatin E (MMAE). The compound is nowadays in use in China as a treatment of gastric cancer [111, 112]. Its effectiveness and safety were determined in a retrospective study in HER2 positive patients with metastatic BC who received therapy outside a clinical trial setting. The results showed a median real-world PFS (rwPFS) of 5.9 months and an ORR of 29.6, which is in agreement with previous clinical trial results. Additionally, there was some evidence that RC48 may have better performance when administered in the initial portion of the treatment regimen, and timing sequence with other medications, such as pyrotinib or capecitabine, had no significant effect on rwPFS [113]. Mechanistically, RC48 has anti-tumor effects (perturbation of downstream HER2 signaling) and is able to deliver cytotoxic loads on cancer cells to eliminate them. RC48 exhibited its equivalent efficacy over FDA-approved, T-DM1 in HER2-positive BC and gastric cancer models including those resistant to trastuzumab and lapatinib in a preclinical study (NCT02881190) [114].

**ARX-788** ARX-788 is a next generation HER2-targeted ADC, which uses a new cytotoxic molecule called amberstatin 269 conjugated to a mAb site. This location-specific connection increases the stability and homogeneity of the ADC, which can increase the effectiveness and safety. It has been demonstrated in clinical studies that ARX-788 demonstrated a significant PFS in individuals with HER2-positive BC that had prior trastuzumab and taxane-based treatments. Although some cases of ophthalmic and interstitial lung-related adverse effects were noted, they were manageable and could be absorbed at acceptable tolerability levels. In addition, even without prophylactic premedications its hematologic and gastric intestinal toxicities were similar to those of the previous-generation ADCs [115].

**ALT-P7** ALT-P7 is a novel HER2-targeting ADC, which consists of two MMAE molecules conjugated to a trastuzumab variant with a cysteine-containing peptide motif, making conjugation site-specific. The design enables greater stability and delivery specifications. To determine the safety, pharmacokinetic, and early efficacy of ALT-P7 in heavily pretreated patients with HER2-positive BC, the first-in-human clinical study was carried out in 2020. The acceptable safety outcomes were achieved as the study set a well-tolerated dose of 4.2 mg/kg. Even though dose-limiting toxicities are being investigated, the clinical activity exhibited justifies the advance of ALT-P7 into phase II trials to be evaluated [116].

**BL-M07D1** BL-M07D1 is an innovative HER2-directed ADC and is made of trastuzumab-based mAbs conjugated using a cathepsin B-cleavable linker to the cytotoxic drug ED-04. ED-04 acts by inducing apoptoticcell death by inhibiting the S-phase of the cell cycle. BL-M07D1 has a high DAR of 8:1 similar to that of T-DXd, and is reported to be more stable. Significantly, its effects on bystanders are high, which makes it most effective when used against tumors containing a population of HER2-positive and HER2-negative cells. These features show that BL-M07D1 is a promising candidate that can overcome the shortcomings currently experienced with the older HER2-targeted therapeutic approaches. It is in a phase I clinical trial with patients with metastatic BC, currently in evaluation phase [117].

**Zanidatamab zovodotin** Zanidatamab zovodotin is a biparatopic ADC which binds two non-overlapping domains of the HER2 receptor, which increases the receptor clustering, internalization, and antitumor effect. In this ADC, biparatopic humanized mAb zanidatamab is conjugated with an auristatin payload conjugated at the end of a protease-cleavable linker [118]. Zanidatamab in particular has been designed to bind two different epitopes to HER2 simultaneously, which enhances its binding avidity and uptake capability. The current phase I first-in-human clinical trial will be used to identify the maximum tolerated dose (MTD), safety and tolerability, and antitumor effectiveness of the monotherapy. Early findings reported a 13% total response rate (ORR) of patients with BC that consisted of 13% partial response rate and 38% stable disease rate. The results show a good safety profile and promising clinical activity, yet stronger clinical trials are required to determine the therapeutic potential and the long-term effectiveness of it [119].

#### TROP-2 targeted antibody–drug conjugates

**Fig. 5:**
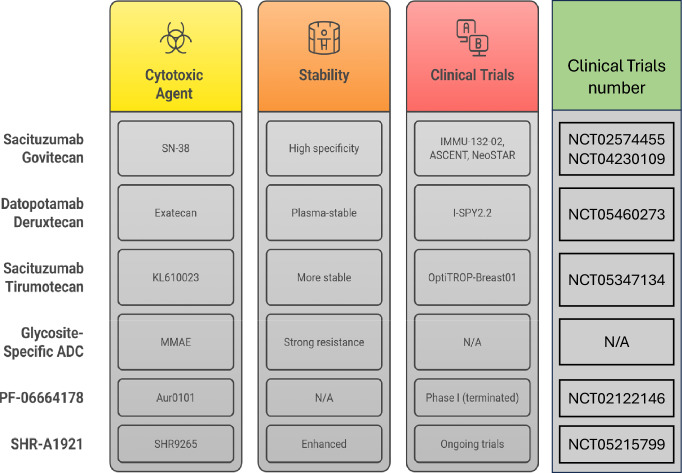
Comparison of key properties and clinical development stages of selected TROP-2 Targeted Antibody–Drug Conjugates. The chart categorizes six ADCs, based on their cytotoxic agent, stability profile, and clinical trial status

The TROP-2 is a transmembrane glycoprotein, with minimum levels of expression in the majority of normal tissues, and a broad spectrum of excessive expression in tumors including BCs. It has been observed to be overexpressed in enhancing tumor progression. Particularly, low TROP-2 levels may condition the progression to TNBC, and big levels are linked to aggressive tumor progression, e.g. increased proliferation, invasive ability, metastatic risk, and resistant to treatment, which highlights its usefulness as a therapeutic target [120]. Figure 5 shows the comparison of important properties studied TROP-2 Targeted ADCs.

**Sacituzumab govitecan (SG)** SG is an advanced TROP-2-targeting ADC approved for clinical use in metastatic TNBC [121]. Notably, SG was the first third-generation ADC to gain regulatory approval and remains the only ADC currently authorized for treating advanced TNBC as well as hormone receptor-positive (HR+)/HER2-negative BCs. It consists of SN-38, an active metabolite of irinotecan, linked to a humanized IgG1 mAb (HRS7). The DAR lies between 7.5 and 8, providing a high payload delivery per antibody. Incorporating improved linkers and potent cytotoxic agents, SG achieves high specificity with reduced toxicity [122].

To date, three major clinical trials have evaluated SG. The first, a Phase I/II trial (IMMU-132–02), enrolled patients with resistant and metastatic TNBC. This was followed by a Phase II/III trial (ASCENT), which used a similar design to the earlier study. Most recently, the NeoSTAR Phase III study concluded in December 2023 and was the first to assess SG in a neoadjuvant setting, before either radical or conservative breast surgery in patients with moderately favorable TNBC prognoses [123, 124].

**Datopotamab deruxtecan (Dato-DXd)** Dato-DXd is another promising TROP-2-directed ADC that combines a humanized IgG1 mAbs against TROP-2 with topoisomerase I inhibitor derived from exatecan. This cytotoxic payload is connected via a tumor-selective, plasma-stable tetrapeptide cleavable linker designed to minimize systemic toxicity and enhance tumor specificity [125]. In a Phase II neoadjuvant I-SPY2.2 trial, employing a sequential multiple-assignment randomized design, Dato-DXd was administered with durvalumab (a PD-L1 checkpoint inhibitor) as a first-line treatment in high-risk Stage 2/3 BC. The preliminary results suggested a potential therapeutic benefit in this early disease setting [126].

**Sacituzumab tirumotecan (SKB264)** SKB264 is a new ADC that uses the HRS7 of SG. The difference however lies in the cytotoxic agent that is being used where KL610023 a belotecan-derived topoisomerase I inhibitor is used. Its DAR (7.4) is similar to that of SG (7.6), but SKB264 exhibits a more defined conjugation chemistry, which can probably limit its toxicity [127]. These preclinical trials showed that the SKB264 half-life is considerably longer in murine models than SG (57 h vs. 14 h), which implies superior pharmacokinetic behaviour [128]. Its Phase III OptiTROP-Breast01 trial (NCT05347134) is underway to determine its efficacy in patients who have a history of advanced, recurrent, or metastatic TNBC who had received at least two prior therapies [129]. The most common reported negative outcomes are neutropenia (23.7%), anemia (20.3%), and thrombocytopenia (16.9%) [130].

**Glycosite-specific ADC (gsADC 3b)** The site-specific versions of ADCs targeting the TROP-2-targeting HRS7 antibody have been engineered as a new generation using either a glycoengineering approach or traceless affinity-mediated approach incorporating MMAE. One-step modification was used to synthesize the glycoengineered construct, gsADC 3b, which forms stable, site-specific ADCs with high aggregation resistance and significant antitumor activity in vivo, providing a start point in TROP-2-targeted therapy [131].

**PF-06664178** PF-06664178 is a respirator TROP-2 site-specific ADC that has been developed by Pfizer. It contains a humanized IgG1 antibody (RN926) linked to a potent auristatin derivative, Aur0101 by an AcLys-VCAur0101 linker, which breaks tubulin polymerization at picomolar concentrations. Experiments in preclinical phase exhibited strong cytotoxic impacts on TROP-2-expressing cancer cells, and strong antitumor impacts were shown in various patient-derived xenograft (PDX) models, like TNBC, lung, and ovarian tumours [132]. In the year 2014, a Phase I trial was initiated to assess the MTD, safety and pharmacokinetics of Pf-06664178 in participants with advanced or metastatic solid tumors. Nevertheless, the study was discontinued prematurely because the toxicity was so great at higher doses that it counterbalanced its therapeutic improvements [133].

**SHR-A1921** SHR-A1921; is a TROP-2-binding ADC consisting of a proprietary humanized IgG1 molecule conjugated to a topoisomerase I inhibitor termed SHR9265 with a cleavable linker. SHR9265 is a further optimization of exatecan with increased lipophilicity and cellular permeability. Preclinical comparative analyses of SHR-A1921 have demonstrated it to be superior to both SG and SKB264 regarding antigen-binding affinity, plasma stability, bystander-killing effect, and general in vivo efficacy and half-life [134]. SHR-A1921 is now in preclinical testing on its therapeutic profile in a number of solid tumors, with the endeavor to evaluate different dosages and cruskible types to further establish the clinical usefulness of the drug.

#### HER3-targeting ADCs

HER3, a member of the ErbB family of receptor tyrosine kinases, plays a key role in activating the PI3K/AKT signaling cascade. Overexpression of HER3 has been linked to resistance against therapies targeting other HER receptors, such as HER2 and EGFR, and is significantly involved in the progression and metastasis of BC [135].

**Patritumab deruxtecan (HER3-DXd)** HER3-DXd (or patritumab deruxtecan) is an anti-HER3 ADC containing an anti-HER3 mAb that is linked to a topoisomerase I inhibitor (DXd) through a cleavable tetrapeptide linker. Cleavage in the TME causes the release of DXd causing DNA damage, arrest in the cell cycle and apoptosis. Moreover, HER3-DXd can induce immune responses and antitumorimmunity [136]. This ADC has a high binding affinity to HER3 and is able to internalize into cancer cells, inducing apoptosis by the release of the cytotoxic payload [137].

In a Phase I/II trial, the ORR was 42.9% in HER2-positive disease (n = 14), 22.6% in TNBC (n = 53) and 30.1% in the HR + /HER2-negative BC (n = 113). In another neoadjuvant trial in which only one dosage of HER3-DXd was investigated, the ADC presented a 45% ORR and increasing scores of tumor-infiltrating lymphocytes and overall antitumor activity. It is worth noting that the response was not correlated with baseline HER3 mRNA expression [138]. Krop et al. reported the safety and clinical efficacy of HER3-DXd in heavily pretreated HER3-positive metastatic BC in the multicenter Phase I/II U31402-A-J101 trial (NCT02980341; JapicCTI-163401). Neutropenia, leukopenia, anemia, and thrombocytopenia were the most frequently occurring adverse events of treatment emergence. ORR 42.9% in HER2-positive, 22.6% in TNBC, 30% in HR + /HER2-negative subgroups were reported [139].

**EV20/MMAF (Monomethyl auristatin F)** EV20/MMAF is an HER3-binding ADC that consists of a humanized antibody EV20 conjugated with cytotoxin MMAF through a non-cleavable linker. This construct has proved to be selectively cytotoxic to HER3-expressing melanoma and BC cell lines. Its favorable preclinical results demonstrated that it is a potent antitumor agent, highly stable and not very toxic thus good prospective developmental candidate drug [140, 141].

#### LIV-1 targeting ADCs

LIV-1 is a transmembrane zinc transporter protein that also possesses metalloproteinase activity and is frequently overexpressed in BC. Its involvement in epithelial-mesenchymal transition (EMT) underscores its relevance as a therapeutic target [142].

**Ladiratuzumab vedotin (SDP03923-000–9106)** The only ADC under clinical trial that targets LIV-1 is ladiratuzumab vedotin (SDP03923-000–9106). It is comprised of a LIV-1-targeting mAb-conjugated to MMAE with a DAR of about 6. In preclinical, high binding affinity and efficient internalization in different cancer cell lines, such as BC, prostate, gastric, and non-small cell lung cancer cells was demonstrated [142].

Even though the initial clinical results indicated low efficacy in the HER2-positive disease, ADC proved to have potential activity in TNBC, with 32.3 percent ORR. Nausea (60%), fatigue (58%), neuropathy (54) and constipation (39) were common adverse events that were associated with treatment. Grade 3 or above recorded neutropenia (21%), fatigue (14%), and metabolic alteration (hyperglycemia, hypokalemia, and hypophosphatemia) (12% each). As of 2019, ladiratuzumab vedotin is undergoing clinical Phase Ib/II trials in combination with an ICI pembrolizumab as first-line treatment of advanced TNBC (SGNLVA-002/KEYNOTE 721) [143].

Safety incidents have been a major and inevitable issue in the clinical context. The toxicity profile of ADCs is dependent on several critical factors such as: the antigen target, the pharmacological activity of the cytotoxic agent, the nature of a linker, and the sites of conjugation. Hematopoietic system, liver, kidney, and lungs are particularly vulnerable to severe forms of toxicity, with medical intervention being necessary [144]. The early discharging of cytotoxic reagents may lead to hematologic adverse effects such as anemia, leukopenia, thrombocytopenia, neutropenia, mostly because of the targeting of high-division cells in liver and bone marrow [145]. In order to control these toxicities, the dosing of ADC should be meticulously adjusted in clinical practice. It is important to closely track blood parameters to identify hematologic adverse effects and provide supportive therapies to increase the level of erythrocyte, platelet, or neutrophil.

One of the major therapeutic concerns with ADCs is maintaining drug efficacy, which is influenced by multiple determinants. The half-life of the conjugate and the design of the linker play pivotal roles in ensuring effective tumor targeting after intravenous administration. A suitable half-life allows the ADC to reach the tumor site efficiently [146], while the linker must be sufficiently stable to prevent premature payload release. Furthermore, cellular internalization is a key step in achieving therapeutic action [147].

### Antibody-conjugated nanoparticles

The ACNPs are a highly adaptable and rapidly developing platform that combines both the selectivity of targeting of mAbs with the versatile physicochemical potential of the nanocarrier. In these systems, there are improved tumor localisation, regulated release of the payload and minimised the systemic toxicities when compared to traditional ADCs. ACNPs enable the movement of antibodies into tissues and overcoming the biological barriers, including non-uniform antigen expression and low tissue penetration [148]. The current trends in polymeric, lipid-based, and hybrid nanostructures have shown better medication delivery and increased therapeutic efficacy in preclinical BC models, and ACNPs are suggested to be the next-generation targeted therapeutics [149]. ACNPs are still investigational and currently have no FDA-approved products; previous and current research involves specialized immunoliposomes that have been tested in early preclinical studies.

ACNPs represent a cutting-edge strategy for precise DDS [150]. These systems combine structural innovation with functional versatility. Two principal techniques, physical adsorption and covalent bonding, are employed to fabricate ACNPs [18]. A diverse array of nanocarriers has been utilized in the development of ACNPs for advanced therapeutic applications (Table 3).

**Table 3:** Antibody-conjugated nanoparticles in breast cancer therapy

Drug/agent	Nanocarrier type	In-vitro/In-vivo model	Outcomes	References
Trastuzumab-Docetaxel	Immunoliposomes	–	Enhanced efficacy compared to free drug, non-conjugated carriers, antibody alone, and T-DM1	[152]
Trastuzumab-loaded LPH NPs	Lipid-polymer hybrid nanoparticles	BT474, MCF7 cells	Higher cytotoxicity in HER2 + BT474 cells vs MCF-7	[154]
Trastuzumab with PLGA NPs	PLGA nanoparticles	MDA-MB-231, MDA-MB-468, MCF-7, T47D, ZR-75–1, BT-474, SKBR3	Dose reduction, increased cytotoxicity, and apoptosis	[155]
Curcumin	Chitosan maleate/thiolated alginate NPs	SK-BR-3 cells	Higher cellular uptake and anti-cancer activity than free curcumin or unconjugated NPs	[156]
Paclitaxel	Polymeric niosomes	–	Greater drug release and tumorsuppression	[157]
Sorafenib + Nutlin-3a	Nylon-11 nanoparticles	SKBR3, HTB-30 cells	Highest cellular uptake with trastuzumab-conjugated formulation	[158]
Paclitaxel + ABCB1-siRNA	Cationic polymeric liposomes	BT-474, MCF-10A; BT-474 tumor-bearing mice	Superior tumor targeting, uptake, and therapeutic efficacy	[159]
Trastuzumab-Zn-Mn-Fe NPs	Magnetic nanoparticles	RPE-1, SK-BR-3, MDA-MB-231	14% viability reduction, synergistic effect, minimized antibody side effects	[160]
Pyrotinib	Mesoporous selenium nanoparticles	SK-BR-3 cells	Induced apoptosis and inhibited proliferation	[161]
DOTA (Chelator)	Mesoporous carbon–silica nanostructure	SK-BR-3, BT-474, MDA-MB-231	Improved HER2-targeted delivery vs non-conjugated variants	[162]
Lonidamine + Trastuzumab	Lipid nanoparticles	–	Synergistic effect, 14 × apoptosis compared to the control group	[163]
Ricin	Immunotoxin nanoparticles	MCF-7 cells	70–78% cell death vs 53–62% with non-encapsulated ricin at 24–48 h	[164]
Bevacizumab-Doxorubicin	177Lu-labeled iron oxide immunoliposomes	4T1, MCF-7, SKBR3, MDA-MB-231; 4T1 tumor-bearing mice	Increased tumor accumulation and prolonged retention	[166]
Bee pollen extract + BSA	Hybrid peptide–protein hydrogel NPs	MCF-7 cells	Potency enhanced synergistically with bevacizumab	[167]
siXBP1 (siRNA)	Polymer-lipid hybrid nanoparticles	MDA-MB-231 cells	Superior gene silencing and anti-cancer effect vs commercial alternatives	[168]

#### Trastuzumab-conjugated nanoparticles

Among various platforms, immunoliposomes have gained notable attention. Owing to their phospholipid bilayer structure, which mimics cellular membranes, immunoliposomes improve the pharmacokinetic characteristics of encapsulated agents. Additional benefits include controlled drug release, biocompatibility, and preferential accumulation at tumor sites [151]. A formulation comprising 140-nm docetaxel-loaded trastuzumab immunoliposomes, developed using maleimide linkers via the thin-film hydration method, demonstrated tumor uptake comparable to that of marketed formulations. However, these immunoliposomes showed superior therapeutic performance both in vitro and in vivo [152].

The design of nanocarriers often involves natural or synthetic polymers, enabling a triphasic drug release profile, initial burst, diffusion, and polymer erosion. Tailoring these release phases through polymeric architecture enhances DDS efficiency [153]. In one study, lipid-polymeric nanoparticles (NPs) with electrostatically adsorbed trastuzumab exhibited selective cytotoxicity against HER2-positive BT474 BC cells, while sparing HER2-negative MCF7 cells [154]. Notably, trastuzumab-loaded PLGA NPs induced greater apoptosis and cytotoxicity than free drug forms. Even at half the dosage, these NPs matched the efficacy of the full dose of free trastuzumab, highlighting their potential in BC therapy [155].

In another approach, chitosan-maleate and thiolated alginate were combined via ionic gelation and verified using click chemistry to synthesize NPs loaded with curcumin and functionalized with maleimide-linked trastuzumab. These targeted curcumin-trastuzumab NPs (Tras-Curcumin-NCs) showed enhanced cytotoxicity and lower IC50 compared to non-targeted formulations, suggesting superior targeting and therapeutic potency. Flow cytometry confirmed a significant increase in apoptosis, positioning Tras-Curcumin-NCs as effective carriers for targeted BC therapy [156].

Additionally, paclitaxel and Fe₃O₄-loaded niosomes functionalized with trastuzumab were incorporated into an electrospun polycaprolactone/chitosan mat for localized BC treatment [157]. Nylon-11 NPs have also emerged as promising candidates, mimicking TME through pH-responsive release profiles. While drug-loaded versions exhibited dose-dependent cytotoxicity, their bare counterparts showed favorable biocompatibility [158]. Moreover, cationic liposomes co-encapsulating paclitaxel and ABCB1-siRNA with trastuzumab have demonstrated therapeutic efficacy in BC models [159].

Mesoporous silica and carbon NPs have attracted considerable interest due to their high surface area, tunable porosity, and excellent biocompatibility. Hollow mesoporous selenium NPs, functionalized with albumin and loaded with pyrotinib, were developed for HER2-positive BC targeting [160]. Likewise, ^177Lu-labeled mesoporous Carbon@Silica structures have been reported for targeted radionuclide therapy in HER2-positive malignancies [161].

While fewer nanocarriers have been reported, promising results include trastuzumab conjugation with novel Zn₀.₄Mn₀.₆Fe₂O₄ NPs achieving an 80% loading efficiency [162], and hybrid lipid NPs encapsulating lonidamine (a hexokinase 2 inhibitor) along with trastuzumab to enhance target specificity. These hybrid systems offer a platform for future modifications to improve chemotherapeutic outcomes [163].

Another innovative strategy involved a ricin-Herceptin immunotoxin built on a chitosan base, which exhibited potent cytotoxic effects. After 24 and 48 h of treatment, the immunotoxins eliminated 70% and 78% of MCF-7 cells, respectively, whereas non-encapsulated forms induced lower cytotoxicity (53% and 62%) under similar conditions [164].

#### Bevacizumab-conjugated immunoliposomes

Bevacizumab, a humanized mAb targeting vascular endothelial growth factor (VEGF), plays a key role in anti-angiogeniccancer therapy [165]. Radiolabeled NPs have emerged as a versatile modality capable of circumventing limitations associated with conventional approaches, offering prospects for individualized treatment in BC. For instance, iron oxide NPs coated with alginic acid, stabilized with PEG, and conjugated with doxorubicin and bevacizumab have been explored [166]. In another study, polyphenolic compounds isolated from bee pollen were encapsulated in peptide-protein hybrid hydrogel NPs formed by bovine serum albumin and protamine sulfate, with folic acid used for targeting lung and BC cells [167].

#### siXBP1-targeted delivery

In the context of TNBC, PLGA lipid nanoparticles conjugated with siXBP1 have been reported to effectively silence the XBP1 gene in MDA-MB-231 cells under hypoxic conditions. These NPs achieved 75% knockdown efficiency and significantly enhanced apoptosis, approximately tripling the rate compared to normoxic conditions. These findings pave the way for further in vivo studies and suggest potential clinical applications in TNBC therapy [168].

ACNPs are proving to be a promising modality in both cancer diagnostics and therapeutics, owing to their ability to enhance drug specificity and minimize systemic toxicity. However, challenges remain, particularly regarding the optimization of linker properties, which influence long-term toxicity, metabolic fate, and cellular uptake. Future investigations should prioritize the design of suitable modifications to enhance delivery efficacy and therapeutic outcomes.

Multiple nanoscale chemotherapies are routinely used in the treatment of BC: albumin-bound paclitaxel (nab-paclitaxel) is approved in metastatic BC and eliminates solvent-related toxicities and pegylated liposomal doxorubicin (PLD; Caelyx/DOXIL) is authorized in Europe in metastatic BC where cardiac toxicity may be a concern [169]. One of the polymeric micelle paclitaxel (Genexol-PM) was non-inferior/superior to traditional paclitaxel in phase III study and is approved in the region (Korea) [170, 171]. Another example, targeted liposomes (e.g., MM-302, HER2-directed PLD) depict a picture of translational challenges, encouraging early results, but failed phase 2, requiring a strong target engagement and trial design.

## Challenges in clinical translation

### Tumor heterogeneity and antigen variability

Tumor heterogeneity, including both intra-tumoral and inter-tumoral variation, greatly limits the practical application of antibody-mediated DDS for BC. Variability in tumor cell populations including genetic abnormalities, receptor expression, and metabolic characteristics impairs the targeting and destruction of cancerous cells [172–174]. Especially difficult, BC is defined by several subtypes, including HER2-positive, hormone receptor-positive, and TNBC, each with specific molecular and antigenic traits [175]. The different profiles result in different antigen expressions, therefore compromising the efficacy of targeted treatments such as ADCs. HER2-positive BC is a good illustration of this difficulty since HER2-targeted treatments such as Trastuzumab are often employed [25, 176]. HER2 expression, however, still varies throughout the tumor mass. Some cancer cells could have lower or absent HER2 expression, which would result in inadequate targeting and treatment failure [177].

Research indicates that this antigenic variation could encourage antigen escape, wherein cancer cells under therapeutic selection pressure downregulate or erase HER2 expression, hence making the surviving tumor cells resistant to treatment [178]. Tumors with different HER2 expression levels in HER2-positive BC show different sensitivity to Trastuzumab, emphasizing the challenges of defining consistent treatment outcomes when concentrating on a single antigen [179]. Furthermore, antigen variability crosses intra-tumoral heterogeneity to metastatic locations, where antigen expression could differ significantly [180]. A particularly difficult subtype of TNBC is one defined by the absence of ERs, PRs, and HER2 expression. Aggressive conduct and a poor prognosis define this subtype, mainly because of the varied expression of tumor antigens at several metastatic sites [181]. In some cases, the expression of TROP-2, a possible target for ADCs like SG, may vary between primary and metastatic lesions [82]. A study by Sakach et al. (2022) found TROP-2 expression to be heterogeneous even among metastatic sites within the same patient, hence reducing the effectiveness of treatment targeting this antigen [182]. This case underlines the difficulty of achieving uniform treatment outcomes in cancers with different antigen patterns spread across several sites [183].

Apart from antigen variety, the TME hinders antibody-based treatment even more. Several Factors like hypoxia, immune cell infiltration, and the extracellular matrix (ECM) may affect antigen expression [184], hence limiting the target availability for ADCs [185, 186]. Decreased oxygen levels start hypoxia-inducible factors (HIFs), which may change the expression of surface antigens and so impede ADC penetration in hypoxic tumor areas. The ECM might also impede antibodies from reaching cancer cells, particularly in solid tumors like BC where dense stromal tissues block the entrance of therapeutic medications. These problems have led to several planned remedies [187, 188]. bsAbs might target two separate antigens at once, therefore preventing antigen escape. Blinatumomab, a bsAb targeting CD19 and CD3, has demonstrated effectiveness in acute lymphoblastic leukemia (ALL) by driving T lymphocytes to combat cancer cells [189]. Similarly, bsAbs might be designed for BC to target many tumor antigens, hence increasing the likelihood of effectiveness in varied tumors. The development of dual payloads in ADC systems is another choice [190].

Targeting several cancer cell types, these ADCs offer two separate deadly compounds to the tumor. This approach can reduce tumor heterogeneity, hence ensuring that the treatment may still target a different antigen or pathway if one antigen is eliminated [191]. SG, which targets TROP-2, demonstrates this approach and shows potential in TNBC, a subtype often marked by varied antigen expression [192].

Personalized medicine, where drugs are tailored to the particular antigen patterns of people's tumors, is expected to shape the future of antibody-mediated therapies in BC therapy [193]. Studying the genetic and antigenic markers in every patient's cancer helps doctors develop customized ADC therapies that target the characteristics of the tumor. Integrating ADCs with different immunotherapeutic plans such as T-cell engagers and ICIs might also have synergistic effects. Utilizing enhanced therapeutic effectiveness and control of antigen variety, these mixed therapies might help the immune system target both antigen-positive and antigen-negative cancer cells [194, 195].

### Immunogenicity and off-target effects

Particularly in BC, immunogenicity and off-target effects significantly restrict the utilization of ADCs. These issues reduce the efficacy of drugs and have obvious side effects, therefore stressing the need of exactly modify antibody-based treatments to make them safer and more powerful [25, 179]. One of the compelling disadvantages of using antibodies in cancer therapy is immunogenicity, the ability of therapeutic antibodies to provoke an immune response [182, 189]. Humanized antibodies like Trastuzumab (Herceptin) may have brought cutting-edge innovation in the treatment of HER2-positive BC.

Contrarily, the risk of immunogenicity remains because immune responses may elicit the creation of neutralizing antibodies apprehending therapeutic efficacy or hastening drug clearance [25, 179]. The immunological responses against a drug, like trastuzumab, may create anti-idiotype antibodies that bind to the therapeutic antibody, thereby counteracting its action. Seldom, this immune reaction might lead to treatment cessation or dose reductionand give patients infusion-related symptoms including fever, chills, and headaches [182, 186]. Because they are more murine than humanized or completely human, therapies utilizing murine mAbs have attracted particular notice regarding this immunogenicity problem as they provoke stronger immune responses [178]. Since the immune system identified these antibodies as alien, early cancer treatments utilizing murine mAbs caused notable allergic responses. Humanizing antibodies, by replacing most of the murine components with human sequences, has lowered immunogenicity [13, 110]. Humanized antibodies can, however, particularly in genetically predisposed people, trigger immunological responses, hence stressing the need to properly control immunogenicity in the creation of novel treatments [185, 195].

Another significant difficulty in cancer treatment with ADCs is off-target consequences. The therapeutic antibody or ADC linking to undesired antigens on normal tissues causes these symptoms, hence causing toxicity. One well-known illustration of this is the HER2-targeted drug Trastuzumab, which also attaches to HER2 receptors on cardiomyocytes, and heart cells in addition to HER2 on BC cells [176, 190]. Patients receiving Trastuzumab show well-documented cardiotoxicity caused by this interaction. HER2's expression in several other organs, including the lungs, ovaries, and kidneys, as well as its presence in cancer cells makes it more difficult to accomplish targeted medication delivery to cancer cells while preserving healthy tissues [150, 187]. Indeed, especially for individuals with pre-existing heart disease, cardiotoxicity is among the main reasons patients on Trastuzumab stop treatment [177, 196].

Researchers looking to solve this are investigating the creation of more selective ADCs that can precisely attach to tumor-specific antigens, hence sparing healthy tissues. Reducing off-target effects by improving targeting specificity is the goal of next-generation ADC development [181, 183]. Improvements in linker technology have been crucial in this initiative, enabling the regulated release of deadly chemicals just inside target cells. For example, cleavable linkers guarantee that the payload is delivered only when the cancer cell internalizes the ADC, hence reducing systemic toxicity [172, 186, 194]. The payload is still important even with the best linkers, though. Certain cytotoxic drugs utilized in ADCs, such as maytansine and MMAE, might nevertheless have off-target effects because of their strong action on dividing cells. The difficulty is yet to guarantee that these chemicals only influence the cancer cells being targeted, hence reducing undesired toxicities [13, 178]. SG, which targets TROP-2 in TNBC, is an intriguing illustration of an optimized ADC showing how linkers and payloads can reduce off-target effects [110, 195]. Minimizing the effect on normal tissues, this ADC employs the chemotherapeutic drug SN-38 triggered within the cancer cell [185]. Still, systematic adverse effects like neutropenia have been noted, indicating that off-target effects in ADC treatment are still a problem [197]. This case emphasizes that even sophisticated ADCs, albeit more selective than their predecessors, nevertheless struggle with systematic toxicities [176, 190]. bsAbs are appearing as a new treatment option to increase tumor specificity even further. By binding to two separate antigens at once, these antibodies help to improve targeting accuracy [150, 187]. Blinatumomab, a bsAb well known, targets CD19 on tumor cells and CD3 on T cells hence activating T cells and causing immune-mediated death of tumor cells [177, 196]. BsAbs provide a method to increase tumor selectivity and lower off-target binding to healthy tissues by interacting directly with the immune system. This bispecific strategy has demonstrated clinical efficacy in treating diseases like ALL and is currently being investigated for more general uses, particularly in BC [181, 183]. Reducing immunogenicity and off-target effects also depends on the use of biomarkers to direct the creation and use of ADCs [172].

Researchers can increase the selectivity of ADCs using the identification and targeting of tumor-specific antigens. Preclinical research using patient-derived xenograft models and genetic profiling helps to more precisely identify targetable tumor antigens, hence guiding the creation of ADCs that uniquely fit the molecular profile of particular tumors. This strategy can improve the general therapeutic effectiveness of ADCs and reduce off-target consequences [186, 194].

### Manufacturing and scale-up limitations

The clinical translation of these potential candidates for BC treatment is greatly affected by manufacturing as well as scale-up constraints of ADCs. Though ADCs show great potential in preclinical and clinical studies, more manufacturing from research settings to large-scale commercial manufacture creates problems compromising the quality and cost-effectiveness of these treatments [198, 199]. The complicated character of ADCs, which are made up of three basic parts: a mAb, a linker, and a cytotoxic payload, causes these disadvantages. Every element requires careful optimization and integration to guarantee the outcome is not only efficient but also safe for patients [200]. Making ADCs has its difficulties; one of them is generating the mAb. Usually utilizing recombinant DNA technology, antibody manufacturing generates significant quantities of the antibody using genetically altered cells, often Chinese hamster ovary (CHO) cells. Maintaining the stability and purity of the antibody while yet achieving positive outcomes is not easy, though [201, 202]. Changes in cell culture conditions, such as temperature, pH, and food availability, can induce variations in antibody quality. For example, glycosylation patterns might differ greatly, which would affect the stability and effectiveness of the antibody and cause batch-to-batch differences [203, 204].

Moreover, raising antibody production from laboratory levels to commercial-scale batches might occasionally need significant bioreactor environmental modifications, hence affecting the quality of the end product. ADCs are more complicated to construct because of their linker component [205]. The linker must be stable in the circulation to avoid early release of the hazardous chemical; it attaches the cytotoxic payload to the antibody. Once the ADC targets the tumor cell, though, the linker has to be cleavable to specifically release the payload [206, 207]. Synthesis of linkers may be somewhat difficult given this fragile balance between stability and cleavability. A technical challenge is guaranteeing that the linkers are properly attached to the antibodies without compromising the integrity of the ADC; the linker chemistry must be repeatable at large volumes [208, 209]. Variability in the conjugation process might cause heterogeneity in the final product, which could lower the therapeutic efficacy and raise the risk of negative consequences. The cytotoxic payloads employed in ADCs are also strong chemicals that must be handled and added into the conjugates cautiously [210, 211]. Highly poisonous are chemotherapeutic drugs like maytansine or MMAE; their inclusion into ADCs must be strictly managed to prevent any exposure to non-target tissues during manufacturing [212]. Precision is required in the process of linking the payload to the antibody as any excess or inadequate loading of the payload can cause suboptimal therapeutic potency or raise the off-target toxicity risk [213, 214]. Maintaining therapeutic action depends on ensuringconstant DAR between ADC batches. Variability in DAR can greatly influence the ADC's effectiveness as larger or lower payload loads can change the drug's capacity to kill cancer cells [215]. Scaling up ADC manufacturing presents difficulties as well in terms of cost-effectiveness. The high cost of producing ADCs is caused by the complexity of the conjugation process, the demand for high-quality antibodies, and the management of hazardous payloads [194, 209]. These elements increase the cost of commercial manufacture of ADCs far more than that of conventional small-molecule medications or mAbs treatments. Particularly in low-resource environments, the costly and labor-intensive manufacturing process might restrict the general availability of ADCs [182, 188].

Moreover, the necessity of specialized production facilities to handle the hazardous loads securely adds further complication to the procedure. Efforts to simplify ADC manufacturing are in progress to solve these problems. Being investigated to improve the efficiency and scalability of antibody production is ongoing bioprocessing, which would enable steadier and higher-yield production [188, 204]. Advances in automated conjugation methods are also being pursued to guarantee more consistent and efficient payload conjugation, hence reducing batch-to-batch variation. Furthermore, the creation of creative linkers and more efficient cytotoxic payloads might simplify the synthesis and production process, hence enabling ADC production to be more affordable and scalable [205, 216].

### Regulatory hurdles and cost considerations

Regulatory hurdles and budgetary constraints significantly influence the development of ADCs for BC therapy as they might postpone the access and availability of these revolutionary therapies [179, 193]. ADCs provide a new set of challenges distinct from traditional small-molecule drugs or even typical mAb therapy by combining the selectivity of mAbs with the power of cytotoxic drugs [186, 190]. Throughout the medication research and commercialization stage, these complexities call for significant economic considerations as well as strict regulatory supervision. The complicated three-part structure of ADCs, the mAb, the linker, and the cytotoxic payload, mostly explains regulatory hurdles in their creation [182, 207]. Every one of these components must be thoroughly examined for safety, stability, and efficacy both in isolation and as part of the ultimate conjugated product [150, 208]. Ensuring ADCs satisfy exacting criteria for purity, strength, and manufacturing consistency falls under regulatory bodies including the U.S. Food and Drug Administration (FDA) and the European Medicines Agency (EMA) [175, 180].

Among the most hotly debated features of ADCs is their linker technology, which guarantees the release of the cytotoxic payload exclusively inside the target cell. A defective linker might cause early release of the dangerous chemical, hence producing off-target effects and harm to healthy tissues, which would greatly lower the therapeutic efficacy of the treatment [203, 214]. Regulatory agencies thus require thorough preclinical and clinical evidence proving that the design of the linker properly regulates payload release, therefore guaranteeing that the medication is active exclusively inside the cancer cells [177]. The rules on the complete structure of ADCs vary more than those of developmental processes in clinical trials. Designing clinical trials for ADCs must consider all histological kinds of BC, including HER2-positive and TNBC. This justifies both safety and effectiveness in a wide range of patients using ADCs [195]. T-DM1 (Kadcyla), for example, is an ADC targeting HER2 that has been under meticulous review for safety and effectiveness. This procedure not only proves that the treatment efficiently targets HER2-positive cells but also that it does not cause too much harm to normal tissues [217]. The licensing procedure of ADCs, considering aspects like the pharmacokinetics, biodistribution, and tumor penetration of the conjugates, is drawn out and complicated by multifactorial regulatory evaluation. Cost issues make it much more difficult to use ADCs in clinical practice [16].

Manufacturing ADCs is a resource-intensive operation; their high manufacturing costs are also influenced by the intricacy of ADC design. Producing high-quality mAbs calls on recombinant DNA technology, usually using CHO cells or other specialized systems, which can be costly [218]. Production costs are further increased by the creation of stable linkers, which must be both stable in circulation and effectively cleavable inside target cells. Moreover, the very effective but costly chemicals employed in ADCs are the cytotoxic payloads like maytansine or MMAE [219]. Specialized equipment and knowledge are needed for the synthesis and precise conjugation of these compounds to the antibody via the linker, hence increasing the total production cost. For example, SG, an ADC aimed against TROP-2 for TNBC, is linked to significant production and therapy expenses, in part because of its SN-38 payload and unique linker technology [220]. This has made the therapy quite costly, hence restricting its availability in underprivileged environments. The price of the finished product generally reflects these high expenses, which can greatly impact the affordability of ADCs for patients, especially in nations with limited healthcare budgets [221].

Furthermore, healthcare systems and insurers place great importance on the cost-effectiveness of ADCs. Though ADCs could be more effective than traditional chemotherapy, their substantial treatment costs usually raise questions from payers and regulatory authorities to guarantee that the extra therapeutic advantages warrant the expense [222]. Manufacturing efficiency and better production methods are being investigated to help to reduce these concerns. To boost output, minimize variability, and save production costs, ADC manufacturing techniques are being linked with continuous bioprocessing and automation [203]. The total cost of ADC treatments might also be greatly lowered by the creation of biosimilars and more reasonably priced payloads, hence enabling access to a larger patient population. Widespread usage of ADC therapeutics will depend on their cost-effectiveness and manufacturing simplification as ADC technology develops [177, 201].

## Future perspectives and directions

### Integration with immunotherapy

Combing immunotherapy-that is ICIs-and ADCs appears promising for enhancing BC therapy. This synergy incorporates various immune activation characteristics of ICIs, for instance, stimulating the immune system of the body in searching for and killing cancer cells, with the precision of ADCs to target and deliver harmful chemicals to tumor cells [223, 224]. This endows treatment efficacy, particularly to BC patients, most especially to some difficult subtypes like TNBC because of the cross-resistance and synergism of these two approaches [225, 226]. ICIs usually allow the tumors to escape immune surveillance by disturbing the relationship between CTLA-4 and PD-1/PD-L1 and with their respective ligands. ICIs thereby can restore T cells to allow the immune system to attack and kill the cancer cells [227, 228]. Anti-PD-1/PD-L1 and anti-CTLA-4 antibodies have especially good outcomes in BC, especially in TNBC, a subtype noted for its aggressive character and lack of targeted treatments [229]. Though there is substantial promise, the immunosuppressive TME and immune escape mechanisms frequently limit the clinical outcomes of ICIs alone [230, 231]. ADCs may be essential in this context. ADCs have an effect, such as T-DM1 (kadcyla) for HER2-positive BC, which directly targets specific antigens using HER2 and provide a means of action as "delivering a cytotoxic payload directly to cancer cells" [232]. Such ADCS not only confer direct killing effectsto cancer cells but also improve the immunogenicity of the tumor when combined with ICIs, thus creating a more favorable environment for immune activation [233]. SG, targeting TROP-2 of ADC, is studied in combination with anti-PD-1/PD-L1 treatments to maximize both direct cytotoxicity and immune activation [82].

The cumulative evidence from preclinical and clinical studies suggests a better therapeutic outcome when the above combination is instigated especially in TNBC patients, usually with a dismal prognosis, since it would lead to increased response rates to therapies, lower chances of immune evasion, and an extended duration of response before relapse [16, 198]. A fine example of the potential of ADC treatment combinations with ICIs is clinical synergy shown through combination treatments involving anti-PD-1 antibodies and HER2-targeted ADCs in HER2-positive BC [176, 231]. In preclinical studies, ADCs aimed at HER2, when used with ICIs, produced improved T cell infiltration and a stronger anti-tumor immune response. Clinical studies are currently looking at this combined strategy to assess its effectiveness and safety in human patients [211, 212]. Likewise, combining PD-L1 inhibitors with T-DM1 and other ADCs has shown potential in improving the immune identification of cancer cells, hence defeating the defense systems of the tumor, and lowering the likelihood of recurrence. Though the preclinical and early-phase clinical findings are encouraging, combining immunotherapy with ADCs presents difficulties [13, 178]. Increased toxicity is one of the main difficulties. Both ADCs and ICIs can alone cause negative consequences; their combination might aggravate these effects, hence causing immune-related negative events (irAEs) or higher off-target toxicity [172, 233]. T-DM1, for example, is linked to cardiotoxicity; combining it with ICis might raise the likelihood of heart damage in patients. The effectiveness of such combination treatments depends on thus, prudent patient selection, dose optimization, and side effect monitoring. Furthermore, important are the financial factors linked to both ICIs and ADCs [177, 205]. Often costing more, these treatments might raise therapy expenses even more when combined, which could hinder their broad use in healthcare systems under financial limits. To justify the cost–benefit ratio, individualized methods to choose the appropriate mix of treatments for the appropriate patient are very essential [206, 223]. Beyond antibody-mediated systems, aptamer-guided delivery strategies are gaining attention for their high specificity and modular design; however, these fall outside the current review’s scope and warrant dedicated discussion elsewhere.

### AI-driven design of antibodies and nanocarriers

The use of AI with antibodies to customize nanocarriers for antibody-mediated DDS seems to be an exciting approach for BC therapy. AI methodologies are increasingly employed to augment the fabrication of nanocarriers and ADCs for improved specificity, stability, and therapeutic efficacy [234, 235]. In the peculiarities of very challenging BC subtypes like TNBC, traditional therapies have been less effective. Therefore, this approach enables a more dedicated attack on the cancer cells [236, 237]. The other major application of AI in antibody design is the production of bsAbs. These antibodies, meant to locate and bind two separate antigens at once, provide the possibility of more exact tumor targeting and immune system activation [238, 239]. AI algorithms model and forecast the best structures for bsAbs, hence guaranteeing both the efficiency and reliability of the dual targeting technique. AI, for instance, has been used in the creation of bsAbs targeting HER2 and ICIs to improve tumor selectivity and defeat immune evasion mechanisms [240]. Using AI, predicting antibody-antigen interactions helps researchers to develop more potent immune-stimulating medications with fewer off-target effects [241].

Apart from antibody optimization, AI is also significantly important in the creation of nanocarriers employed in DDS [242]. Cytotoxic medications are enclosed in nanocarriers such as liposomes, polymeric NPs, and magnetic NPs, which then target them, especially tumor cells [243]. Often, the design of these nanocarriers calls for sophisticated simulations to forecast their size, surface charge, and drug release characteristics. AI algorithms can optimize the properties of nanocarriers for improved tumor penetration and medication release using analysis of great volumes of data from in vitro and in vivo studies [244]. AI, for example, has been utilized to maximize the lipid content of liposomal NPs, hence enhancing their stability and targeting efficacy in HER2-positive BC. Moreover, AI-driven methods help to maximize the linker technology employed in ADCs [245]. The linker is crucial for maintaining the ADC stability in the circulation even as it ensures that the cytotoxic payload is only released within the target cell. AI can assist in extending the therapeutic window of ADCs and lower the toxicity to healthy tissues by forecasting the stability and cleavability of many linkers under specific conditions [246, 247]. AI can also forecast possible immune reactions to the antibodies, hence assisting in the creation of more stable therapeutic proteins with immunogenicity. AI's inclusion into nanomedicine is also spurring the creation of more intelligent nanocarriers able to monitor the medication release process in real-time [248]. In personalized medicine, where knowing the individual TME is vital for customizing DDS, this is especially relevant [249]. By including data from patient-specific biomarkers, AI may create adaptable nanocarriers that react dynamically to the tumor's alterations throughout therapy [250]. Furthermore, clinical decision-making is another developing field using AI. By predicting how different cancer kinds will react to several ADC formulations, AI models let doctors choose more educated treatment plans. Improving efficacy and safety, this strategy can result in the creation of individualized ADCs customized for certain patients [251].

### Personalized antibody-targeted delivery based on biomarker profiling

Particularly when paired with ADCs, personalized antibody-targeted delivery relying on biomarker analysis has surfaced as a novel strategy in the management of BC [226]. Personalized medicine is the idea that pharmaceuticals should be tailored to each patient based on their particular genetic, molecular, and biomarker profiles, therefore guaranteeing that the therapy is both more potent and less risky [233]. Since the illness is somewhat complex and various subtypes may react to treatments differently, this strategy is particularly relevant in BC [201, 210, 233]. Selecting patients depending on certain tumor biomarkers is one of the major developments in individualized antibody-targeted treatment [219, 225]. Finding the most appropriate therapies for patients depends on biomarkers including HER2 in HER2-positive BC, TROP-2 in TNBC, and ER status [195, 217]. In HER2-positive BC, the humanized mAb trastuzumab (Herceptin) is quite effective [16, 198]. Conversely, HER2-negative cancers do not react to this therapy. Biomarker profiling therefore helps to eliminate needless therapies and lower the probability of negative effects by letting doctors choose just those patients who might gain from HER2-targeted treatment [243].

Combining biomarker studies with ADCs enables one to more precisely target cancer cells. Comprising a mAb targeting a particular tumor antigen, a cytotoxic payload, and a linker joining the two, ADCs are healthcare professionals can choose patients for ADC treatment by finding tumors with high amounts of the target antigen, so guaranteeing that the cytotoxic medication is sent exactly to the cancer cells[191, 238, 247]. In malignancies like TNBC, where targeting a particular antigen like TROP-2 has shown encouraging effects with ADCs such as SG, this is very crucial [199]. Selecting patients according to their TROP-2 expression helps to more efficiently direct ADCs to the cancer cells, hence enhancing the therapeutic result and reducing off-target damage [202].

Liquid biopsies are yet another example of biomarker-driven individualization in ADC treatment. Liquid biopsies allow one to discover circulating tumor DNA (ctDNA), which can offer real-time analysis of the genetic profile of the cancer and its changing resistance mechanisms [195, 217]. This method especially helps to track ADC effectiveness over time and see resistance development. For instance, liquid biopsy can monitor HER2 expression variations over T-DM1 treatment in HER2-positive BC, hence enabling therapeutic modifications depending on the tumor response [16, 198, 243]. Furthermore, by directing the choice of ADCs, biomarker research helps to forecast patient treatment reactions as well. Studies have revealed that the success of ADCs is significantly predicted by pre-treatment tumor antigen expression. HER2 expression levels, for example, correspond with the effectiveness of HER2-targeted ADCs such as T-DM1 [191, 247]. While those with low or zero HER2 expression may not have the same therapeutic advantages, patients with tumors expressing high levels of HER2 are more likely to gain from ADC treatment [202, 238].

Likewise, for TNBC, those with high TROP-2 expression are more likely to react to SG, hence highlighting the need for biomarker-driven patient classification. Comprehensive genetic analysis and the integration of AI-driven technologies to more precisely forecast patient reactions to ADCs will shape the future of personalized antibody-targeted delivery [192, 199]. Improvements in liquid biopsy technology and next-generation sequencing (NGS) will enable more exact detection of tumor biomarkers and tracking of therapy effectiveness. Moreover, the combination of many biomarker-driven treatments, such as ADCs with ICIs, is probably going to improve therapeutic results by targeting the heterogeneity of the tumor and immune evasion mechanisms [193, 209].

### Clinical trial harmonization for combination therapies

Advancing antibody-mediated targeted DDS, especially ADCs, in the treatment of BC depends on clinical trial harmonization for combination medicines, which is vital [252, 253]. Combining ADCs with other treatment modalities, including ICIs, has great potential and has drawn much attention. However, the safe, effective, and regulatory acceptance of these treatments in clinical trials depends on their careful harmonization [254]. A strategic plan meant to improve the tumor-killing impact by both directly targeting cancer cells and simultaneously strengthening the body's immune response is the combination of ADCs with immunotherapy, especially ICIs [255, 256]. Combining anti-PD-1 inhibitors with T-DM1 (Kadcyla), an ADC targeting HER2-positive BC, is one example being investigated to use the lethal effects of ADCs along with ICI to lower immune suppression in the TME [257].

Although early-phase clinical studies have produced encouraging outcomes, their effectiveness is mostly dependent on trial protocol standardization to assess the synergy between these two treatment modalities [258, 259]. Harmonizing clinical trials for combination medicines presents several difficulties, particularly in terms of the various mechanisms of action of the medications involved. While ICIs operate by reactivating immune cells, enabling the immune system of the body to assault tumor cells, ADCs function by sending cytotoxic chemicals directly to cancer cells [260]. It is essential to define exact inclusion criteria for patients, monitor probable unfavorable interactions, and ensure the appropriate dosing regimens as these therapies operate via several pathways. For example, T-DM1 might call for particular timing and dose modifications when taken with ICIs to minimize overlapping toxicities such as neutropenia or immune-related adverse events (irAEs), which are prevalent with ICIs [261, 262].

Furthermore, clinical trial harmonization depends on consistent endpoint specification. Often used to evaluate the effectiveness of combination treatments are common goals such as ORR, OS, and PFS [263]. However, it is crucial to make sure these endpoints properly reflect the particular response patterns seen in such combinations when combining ADCs with ICIs. Some cancers, for instance, could have delayed immune-related responses that are not instantly seen in the conventional tumor-shrinking endpoints, hence requiring the use of more thorough monitoring techniques like immune-related PFS (irPFS) [264]. Another important factor that must be harmonized across studies is patient categorization. Identifying biomarkers that forecast a favorable reaction to the mix of ADCs and ICIs would assist in enhancing treatment results and prevent needless damage. Though PD-L1 expression can be a key predictor for ICIs, HER2 overexpression in HER2-positive BC patients is a consistent biomarker for T-DM1 therapy [265, 266]. Combining these treatments in individuals with particular biomarker profiles, such as high HER2 expression and PD-L1 positive [267], is anticipated to produce superior results, hence reducing the chance of non-response or negative consequences [268].

Moreover, the fast creation and approval of combination medications are governed by worldwide clinical trial harmonization. Different regulatory bodies, like the FDA, EMA, and Japanese Pharmaceuticals and Medical Devices Agency (PMDA), could have varying criteria and procedures for combination treatment research [269]. Consistent regional policies will help to speed the creation of combination medicines and provide patients access to these therapies all around [270]. Aligning clinical trial standards, lowering variability, and guaranteeing that studies are meant to provide clinically relevant outcomes all depend on harmonized guidelines as those established by the International Council for Harmonisation (ICH) [271].

Moreover, future versions of antibody-based delivery systems could be combined with immunotherapies, e.g., ICIs, cytokine adjuvants, etc., to increase antitumor effect. Though the discussion of these combinatorial methods is out of the scope of this review, these methods of approach are a bright future in BC therapeutics.

## Conclusion

Antibody-mediated targeted DDS represents a major shift in BC treatment by providing a potent combination of specificity, efficacy, and reduced systemic toxicity. As this article highlights, ADCs and ACNPs have shown significant clinical benefits, particularly in HER2-positive and TNBC subtypes. The evolution of ADCs, from first-generation constructs to sophisticated agents like T-DXd and SG, underscores the influence of linker development chemistry, payload potency, and antigen targeting. Simultaneously, the integration of nanotechnology has broadened the scope of antibody-functionalized delivery platforms, enabling precise payload release and enhanced tumor accumulation.

Despite these advancement, numerous obstacles remain, including tumor antigen heterogeneity, immunogenicity, and off-target toxicity. The therapeutic efficacy of these platforms is often compromised by variable antigen expression and the dynamic TME. Furthermore, immune responses against therapeutic antibodies and unintended interactions with normal tissues remain critical hurdles to clinical translation. Future strategies focusing on bsAbs, dual-payload ADCs, and multi-antigen targeting, along with the incorporation of patient-specific molecular profiling, are expected to refine treatment precision and overcome resistance mechanisms.

While sustained progress will depend on mitigating slowimaging kinetics, tumor heterogeneity, and ADC-related toxicities through smarter design and biomarker-guided use.

## Supplementary Information

Additional file 1.

## Data Availability

No datasets were generated or analysed during the current study.
