# Supplementary material for: Clinical advances and challenges of antibody-mediated targeted drug delivery in breast cancer therapeutics
Source: Discov Oncol. 2026 Feb 1;17:377. doi: 10.1007/s12672-026-04492-5 (PMC12953841; doi:10.1007/s12672-026-04492-5)
Supplement: Supplementary file 1 — Additional file 1. [file 12672_2026_4492_MOESM1_ESM.docx]

**Table 2.** HER2 and TROP2 Targeting ADCs.

| **ADC Name** | **Monoclonal Antibody** | **Linker** | **Payload** | **Clinical Phase** | **Advantages** | **Limitations** | **References** |
| --- | --- | --- | --- | --- | --- | --- | --- |
| Trastuzumab emtansine (T-DM1) | Trastuzumab | Non-cleavable linker (SMCC) | DM1 (microtubule inhibitor) | Approved | Prolonged release, better survival rates, reduced toxicity compared to lapatinib-capecitabine | Fatigue, elevated liver enzymes, thrombocytopenia | [1-4] |
| Trastuzumab deruxtecan (T-DXd) | Trastuzumab | Cleavable GGFG peptide linker | Topoisomerase I inhibitor (DX-8951) | Approved | Delayed drug release, higher efficacy than T-DM1, longer duration of clinical benefit | Interstitial lung disease, pneumonitis | [5, 6] |
| Trastuzumab duocarmazine | Trastuzumab | Cleavable Val-Cit linker | DNA-alkylating agent (Duocarmycin) | Phase I/II | Demonstrated efficacy with tolerable safety | Frequent eye-related side effects | [7, 8] |
| Distamab Vedotin (RC48) | Hertuzumab | Cleavable Val-Cit-PABC linker | Monomethyl auristatin E (MMAE) | Phase I | Higher HER2 binding than trastuzumab, strong antitumor activity | Hematologic toxicity | [9-11] |
| ARX-788 | ARX269 | Non-cleavable para-acetyl phenylalanine linker | Tubulin inhibitor (Amberstatin 269) | Phase II/III | Extended progression-free survival | Ocular and pulmonary toxicity, hematological and GI issues | [12] |
| ALT-P7 | Trastuzumab analog (HM2) | Cleavable disulfide-containing peptide | MMAE (dual payload) | Phase I | High tolerability | Myalgia, sensory neuropathy, alopecia, neutropenia | [13] |
| BL-M07D1 | Trastuzumab | Cathepsin B-sensitive linker | Camptothecin-based inhibitor (ED-04) | Phase I | Effective in both low and high HER2 expression models | – | [14] |
| Zanidatamab zovodotin | Zanidatamab | Cleavable Citrulline linker | Auristatin toxin | Phase II | Partial and stable responses observed | – | [15] |
| Sacituzumab govitecan | HRS7 | Cleavable CL2A linker | SN-38 (active irinotecan metabolite) | Phase III | Enhanced targeting, improved precision | Neutropenia, nausea, diarrhea, fatigue | [16, 17] |
| Datopotamab deruxtecan | Datopotamab | Cleavable tetrapeptide linker | Topoisomerase I inhibitor (Exatecan) | Phase II | Lower systemic toxicity, high selectivity | Stomatitis, one case of interstitial lung disease | [18, 19] |
| Sacituzumab tirumotecan | HRS7 | Pyrimidine-modified cleavable linker | Topoisomerase I inhibitor (KL610023) | Phase III | Improved stability and half-life vs. sacituzumab govitecan | Neutropenia, anemia, thrombocytopenia | [20] |
| gsADC 3b | IgG1 mAb | – | MMAE (microtubule inhibitor) | – | Better solubility and single-step synthesis | Reduced efficacy in dual-step production | [21] |
| PF-06664178 | RN926 | AcLys-Val-Cit linker | Aur0101 (tubulin inhibitor) | Phase I | Stable conjugate | High-dose toxicity | [22] |
| SHR-A1921 | IgG1 mAb | Cleavable tetrapeptide linker | Topoisomerase I inhibitor (SHR9265) | Phase I | Enhanced stability, bystander effect, improved half-life | Nausea, oral mucositis, anemia | [23] |
| U3-1402 | Patritumab | Tetrapeptide cleavable linker | Topoisomerase I inhibitor (DX-8951) | Phase I/II | Induces apoptosis by inhibiting DNA replication | Neutropenia, anemia, thrombocytopenia | [24, 25] |
| EV20/MMAF | EV20 | Non-cleavable linker | MMAF (microtubule inhibitor) | Preclinical | Robust anticancer activity | – | [26] |
| Ladiratuzumab vedotin | Ladiratuzumab | Protease-cleavable linker | Topoisomerase I inhibitor | Phase II/III | Good solubility and cellular uptake | Nausea, neuropathy, fatigue, appetite loss | [27] |

**References:**

[1] Jin KT, Lan HR, Chen XY, Wang SB, Ying XJ, Lin Y, et al. Recent advances in carbohydrate-based cancer vaccines. Biotechnology letters. 2019;41:641-50.

[2] Giugliano F, Corti C, Tarantino P, Michelini F, Curigliano G. Bystander effect of antibody-drug conjugates: fact or fiction? Current oncology reports. 2022;24:809-17.

[3] Diéras V, Miles D, Verma S, Pegram M, Welslau M, Baselga J, et al. Trastuzumab emtansine versus capecitabine plus lapatinib in patients with previously treated HER2-positive advanced breast cancer (EMILIA): a descriptive analysis of final overall survival results from a randomised, open-label, phase 3 trial. The Lancet Oncology. 2017;18:732-42.

[4] Lyseng-Williamson KA. Trastuzumab Emtansine: A Review of Its Adjuvant Use in Residual Invasive HER2-Positive Early Breast Cancer. Drugs. 2020;80:1723-30.

[5] Yan H, Yu K, Zhang K, Liu L, Li Y. Efficacy and safety of trastuzumab emtansine (T-DM1) in the treatment of HER2-positive metastatic breast cancer (MBC): a meta-analysis of randomized controlled trial. Oncotarget. 2017;8:102458-67.

[6] Indini A, Rijavec E, Grossi F. Trastuzumab Deruxtecan: Changing the Destiny of HER2 Expressing Solid Tumors. International journal of molecular sciences. 2021;22.

[7] Cortés J, Kim S, Chung W, Im S, Park Y, Hegg R, et al. LBA1 Trastuzumab deruxtecan (T-DXd) vs trastuzumab emtansine (T-DM1) in patients (Pts) with HER2+ metastatic breast cancer (mBC): Results of the randomized phase III DESTINY-Breast03 study. Annals of Oncology. 2021;32:S1287-S8.

[8] Xu Z, Guo D, Jiang Z, Tong R, Jiang P, Bai L, et al. Novel HER2-Targeting Antibody-Drug Conjugates of Trastuzumab Beyond T-DM1 in Breast Cancer: Trastuzumab Deruxtecan(DS-8201a) and (Vic-)Trastuzumab Duocarmazine (SYD985). Eur J Med Chem. 2019;183:111682.

[9] Turner N, Saura C, Aftimos P, van den Tweel E, Oesterholt M, Koper N, et al. Trastuzumab Duocarmazine in Pretreated Human Epidermal Growth Factor Receptor 2-Positive Advanced or Metastatic Breast Cancer: An Open-Label, Randomized, Phase III Trial (TULIP). Journal of clinical oncology : official journal of the American Society of Clinical Oncology. 2025;43:513-23.

[10] Wang K, Xu T, Wu J, Yuan Y, Guan X, Zhu C. Real-world application of disitamab vedotin (RC48-ADC) in patients with breast cancer with different HER2 expression levels: efficacy and safety analysis. Oncologist. 2024.

[11] Li C, Sun L, Liu Z, Sun H, Wang X, Yu Q, et al. Efficacy and safety of disitamab vedotin after trastuzumab for HER2 positive breast cancer: a real-world data of retrospective study. American journal of cancer research. 2024;14:869-79.

[12] Li Y, Zhang J, Cai Z, Gao X, Zhang L, Lu Z, et al. Disitamab Vedotin (RC48) for HER2-positive advanced breast cancer: a case report and literature review. Frontiers in oncology. 2023;13:1286392.

[13] Tripathy D, Ali K, Agrawal L, Ali SM, Blau S, Block M, et al. ACE-BREAST-03: A phase 2 trial evaluating ARX788, an anti-HER2 antibody drug conjugate (ADC), for the treatment of HER2+ metastatic breast cancer (mBC) in patients who have been previously treated with trastuzumab deruxtecan (T-DXd). Journal of Clinical Oncology. 2024;42.

[14] Park YH, Ahn HK, Kim J-Y, Ahn JS, Im Y-H, Kim S-H, et al. First-in-human phase I study of ALT-P7, a HER2-targeting antibody-drug conjugate in patients with HER2-positive advanced breast cancer. Journal of Clinical Oncology. 2020;38.

[15] Bartsch R. Next generation of drugs in breast cancer. memo-Magazine of European Medical Oncology. 2024;17:280-6.

[16] Sultana R, Chen S, Lim EH, Dent R, Chowbay B. Efficacy and safety of sacituzumab govitecan Trop-2-targeted antibody-drug conjugate in solid tumors and UGT1A1*28 polymorphism: a systematic review and meta-analysis. BJC reports. 2024;2:85.

[17] Goldenberg DM, Sharkey RM. Sacituzumab govitecan, a novel, third-generation, antibody-drug conjugate (ADC) for cancer therapy. Expert opinion on biological therapy. 2020;20:871-85.

[18] Bardia A, Krop IE, Kogawa T, Juric D, Tolcher AW, Hamilton EP, et al. Datopotamab Deruxtecan in Advanced or Metastatic HR+/HER2- and Triple-Negative Breast Cancer: Results From the Phase I TROPION-PanTumor01 Study. Journal of clinical oncology : official journal of the American Society of Clinical Oncology. 2024;42:2281-94.

[19] Shatsky RA, Trivedi MS, Yau C, Nanda R, Rugo HS, Davidian M, et al. Datopotamab-deruxtecan plus durvalumab in early-stage breast cancer: the sequential multiple assignment randomized I-SPY2.2 phase 2 trial. Nature medicine. 2024;30:3737-47.

[20] Cheng Y, Yuan X, Tian Q, Huang X, Chen Y, Pu Y, et al. Preclinical profiles of SKB264, a novel anti-TROP2 antibody conjugated to topoisomerase inhibitor, demonstrated promising antitumor efficacy compared to IMMU-132. Frontiers in oncology. 2022;12:951589.

[21] Xu B, Yin Y, Fan Y, Ouyang Q, Song L, Wang X, et al. Sacituzumab tirumotecan (SKB264/MK-2870) in patients (pts) with previously treated locally recurrent or metastatic triple-negative breast cancer (TNBC): results from the phase III OptiTROP-Breast01 study. Journal of Clinical Oncology. 2024;42.

[22] King GT, Eaton KD, Beagle BR, Zopf CJ, Wong GY, Krupka HI, et al. A phase 1, dose-escalation study of PF-06664178, an anti-Trop-2/Aur0101 antibody-drug conjugate in patients with advanced or metastatic solid tumors. Investigational new drugs. 2018;36:836-47.

[23] He N, Yang C, Yang Y, Xue Z, Xu J, Zhao L, et al. Abstract LB030: SHR-A1921, a novel TROP-2 ADC with an optimized design and well-balanced profile between efficacy and safety. Cancer research. 2023;83:LB030-LB.

[24] Yu HA, Baik C, Kim DW, Johnson ML, Hayashi H, Nishio M, et al. Translational insights and overall survival in the U31402-A-U102 study of patritumab deruxtecan (HER3-DXd) in EGFR-mutated NSCLC. Annals of oncology : official journal of the European Society for Medical Oncology. 2024;35:437-47.

[25] Hashimoto Y, Koyama K, Kamai Y, Hirotani K, Ogitani Y, Zembutsu A, et al. A Novel HER3-Targeting Antibody-Drug Conjugate, U3-1402, Exhibits Potent Therapeutic Efficacy through the Delivery of Cytotoxic Payload by Efficient Internalization. Clinical cancer research : an official journal of the American Association for Cancer Research. 2019;25:7151-61.

[26] Capone E, Lamolinara A, D'Agostino D, Rossi C, De Laurenzi V, Iezzi M, et al. EV20-mediated delivery of cytotoxic auristatin MMAF exhibits potent therapeutic efficacy in cutaneous melanoma. Journal of controlled release : official journal of the Controlled Release Society. 2018;277:48-56.

[27] Zeng H, Wang W, Zhang L, Lin Z. HER3-targeted therapy: the mechanism of drug resistance and the development of anticancer drugs. Cancer drug resistance (Alhambra, Calif). 2024;7:14.
